# Upcycled Orange Peel Ingredients: A Scoping Review on Phytochemical Composition, Extraction Techniques, and Biorefinery Strategies

**DOI:** 10.3390/foods14213766

**Published:** 2025-11-03

**Authors:** Ana A. Vilas-Boas, Daniela Magalhães, Ricardo Gómez-García, Débora A. Campos, Marta Correia, Manuela Pintado

**Affiliations:** 1 CBQF—Centro de Biotecnologia e Química Fina—Laboratório Associado, Escola Superior de Biotecnologia, Universidade Católica Portuguesa, Rua Arquiteto Lobão Vital 172, 4200-374 Porto, Portugal; avboas@ucp.pt (A.A.V.-B.); dmagalhaes@ucp.pt (D.M.); dcampos@ucp.pt (D.A.C.); mmcorreia@ucp.pt (M.C.); 2 CIICYT—Centro de Investigación e Innovación Científica y Tecnológica, Universidad Autónoma de Coahuila, Avenida 3, Esquina con Avenida 16, Colonia Lourdes, Saltillo 25070, Coahuila, Mexico

**Keywords:** phenolic compounds, pectin, essential oils, carotenoids, circular economy

## Abstract

**Background/Objectives**: Orange peels (OP), a major by-product of the juice industry, are rich in bioactive compounds (phenolic compounds, pectin, carotenoids, and essential oils). Its valorization represents a promising route to reduce food waste and foster a circular bioeconomy. This review aimed to map scientific progress in OP upcycling, focusing on the extraction of bioactive ingredients for human nutrition and integrated biorefinery approaches aligned with zero-waste principles. **Methods**: A bibliometric analysis and a scoping review were conducted covering studies published between 2003 and 2023. Scopus database and VOSviewer was usedto identify research trends, hotspots, and gaps. Conventional and emerging green extraction methods were critically compared, and integrated biorefinery strategies for maximizing OP valorization were systematically assessed. **Results**: The analysis revealed an exponential rise in OP research over the past decade, reflecting growing interest in sustainable food waste valorization. Polyphenol- and pectin-rich extracts are currently the focus of research and applications, driven by their high economic and nutritional value. Innovative multi-extraction and zero-waste biorefinery models have emerged, yet most remain at low technological readiness levels. Carotenoids and other bioactive compounds remain underexplored, and challenges persist regarding standardization and scalability. **Conclusions**: OP valorization is shifting towards integrated green extraction and biorefinery frameworks that address clean-label demands, promote circular economy goals, and align with the Sustainable Development Goals. Future research should prioritize (i) standardized protocols, (ii) scalable green extraction technologies, (iii) the inclusion of underutilized compounds such as carotenoids, and (iv) regulatory pathways to accelerate industrial translation.

## 1. Introduction

**Figure 1:**
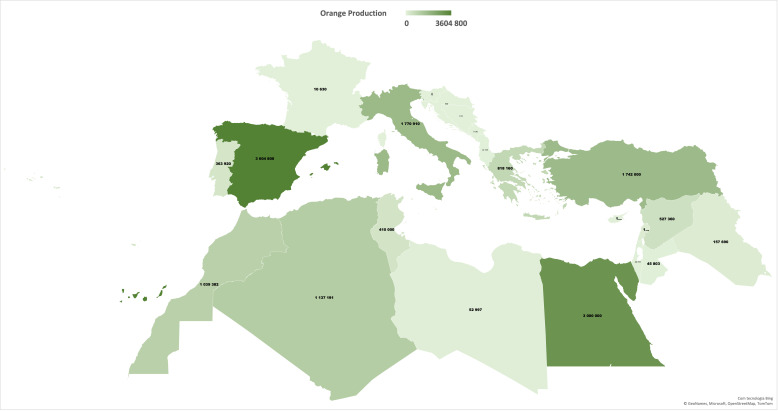
The production of sweet oranges in the Mediterranean basin (in tons) in 2022.

Sweet orange (*Citrus sinensis* L. Osbeck) is one of the most cultivated and consumed citrus fruits globally. It is valued for its taste, nutritional benefits, and health-promoting properties [1,2]. In 2023, global orange production reached 46 million tons [3], accounting for 60% of citrus output. Mediterranean countries are among the most important producing regions (≈12 million tons in 2022). Figure 1 illustrates the distribution of orange production across Mediterranean countries, highlighting the high production in Spain, Egypt, and Turkey. The hot summers and mild winters with marked temperature fluctuations foster superior color and flavor, underpinning the region’s reputation for high-quality oranges [4]. Within the Mediterranean, the European Union (EU) accounts for 47% of output (5.7 million tons in 2022/2023) [5]. Portugal is the fifth largest producer in the EU, with total production reaching 0.38 million tons in 2022. About 74% of Portugal’s citrus area and 90% of the output are in the Algarve. Oranges, clementines/mandarins, and lemons from this region are protected under the EU PGI “*Citrinos do Algarve*”, which has a significant economic impact both nationally and internationally.

According to the statistical data reported by the FAO [6], a large quantity of oranges are processed for juice production, resulting in significant by-products, especially orange peels (OP), which makes up to 50% of the fruit’s weight [7]. These by-products are produced in substantial volumes due to large-scale cultivation and processing practices. Despite their richness in bioactive compounds (BCs) such as essential oils (EOs), phenolic compounds, carotenoids, and pectin, OPs are often discarded through inadequate practices such as landfilling or incineration, creating significant environmental, economic, and social challenges [8,9]. In fact, mismanagement of OP contributes to severe ecological impacts. It has been estimated to account for up to 6% of global greenhouse gas (GHG) emissions when combined with other waste sources [10]. Although OP is sometimes repurposed as animal feed, logistical barriers and limited value added restrict its broader use. To mitigate these challenges, the EU has implemented legislation and strategies, including Directive (EU) 2018/851 and the EU Farm to Fork Strategy, which promotes food waste reduction and upcycling practices, aligning with the United Nations’ 2030 Agenda for Sustainable Development. These regulations aim to protect human and environmental health while creating new business models that capitalize on waste as a biomass source for high-value products, including food ingredients. Replacing the inefficient linear economy, newer frameworks such as the circular economy and zero-waste principles are now central to the EU’s waste hierarchy strategy, driving sustainable food waste upcycling.

There is a growing interest in OP as a research subject, as it is a significant natural source of BCs with proven health benefits and diverse applications. Several upcycled ingredients—food ingredients derived from by-products rather than discarded as waste—have been developed mainly in the form of extracts and powders, offering nutritional and bioactive benefits through innovative food formulations. Currently, upcycled ingredients are developed using sustainable or green chemistry-based techniques that outperform traditional methods, aligning with circular economy principles while reducing time, energy consumption, and environmental impact [11]. However, despite their apparent advantages, the transition to pilot or industrial scale remains limited, as high operational costs often outweigh sustainability goals. Consequently, despite being environmentally unsustainable, conventional methods are still widely used, prioritizing economic feasibility over ecological responsibility. Additionally, the same by-product can be valorized through various upcycling strategies, allowing for the recovery of various value-added ingredients [12]. Recently, innovative valorization strategies have been proposed for OP, including the development of integrated, zero-waste biorefinery-based processes that sequentially recover multiple BCs. These integrated approaches are gaining attention for their nutritional, environmental, economic, and safety advantages over simple standalone extractions [13], and are considered promising pathways to accelerate the transition to a circular bioeconomy that generates co-products instead of waste [14]. Aligned with green chemistry principles, these processes allow for greater extraction efficiency, selectivity, and sustainability.

Growing consumer awareness of natural and health-promoting products has driven interest in OP valorization as a novel source of prophylactic and therapeutic ingredients [15]. Specifically, the consumption of orange flavonoids and pectin has been linked to a reduction in chronic low-grade inflammation, oxidative stress, and intestinal dysbiosis [16,17,18]. Notably, in 2024, the European Food Safety Authority (EFSA) approved glucosyl hesperidin, a major phenolic compound in OP, as a novel food ingredient under Regulation (EU) 2015/2283, reinforcing its potential in nutraceutical applications [19]. Scientific progress and favorable legislation enable the development and commercialization of upcycled ingredients from OP that combine health benefits with food waste reduction and industrial innovation. Numerous studies have explored the extraction, bioactivities, and applications of upcycled OP ingredients, highlighting their potential in establishing new circular economy models. However, to date, no comprehensive review has focused exclusively on OP to critically assess the impact of extraction methods on the quality and functionality of upcycled ingredients. In addition, there is a lack of recent reviews evaluating integrated and zero-waste biorefinery approaches specifically applied to OP valorization. Furthermore, no review has yet provided an in-depth analysis of the therapeutic and prophylactic potential of OP-derived ingredients in the context of gastrointestinal and cardiometabolic health—two areas of increasing public health concern. Therefore, an updated and focused review on these emerging topics is both timely and necessary.

This review presents a scoping review that maps the most relevant scientific advances, trends, and gaps regarding OP upcycling between 2013 and 2023. The review focuses on extraction techniques for the recovery of BCs and integrated, zero-waste biorefinery-based processes to develop upcycled ingredients. To our knowledge, this is the first scoping review dedicated exclusively to OP, combining bibliometric analysis with a critical synthesis of extraction and biorefinery strategies. By identifying current limitations and opportunities, this work provides a comprehensive framework to guide future research and supports the transition towards sustainable, health-oriented circular economy models.

## 2. Bibliometric Analysis

### 2.1. Research Methodology

**Figure 2:**
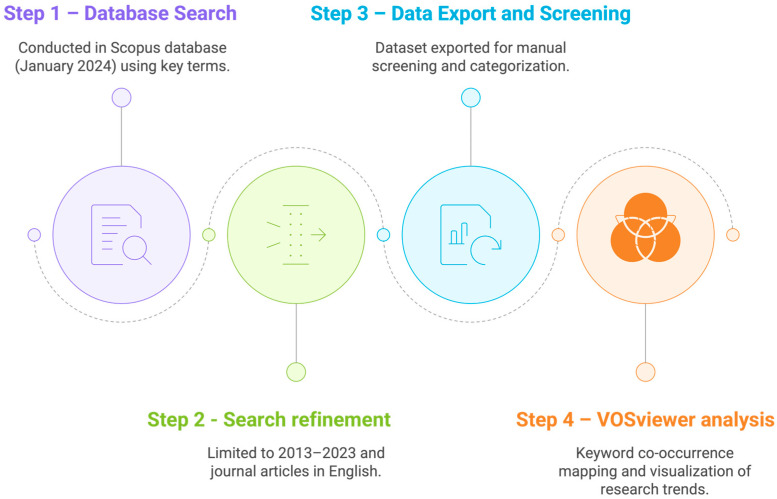
Flowchart of procedure followed in bibliometric analysis (January 2024).

The bibliometric analysis was conducted in the Scopus database in January 2024, considering a time span of two decades (2003–2023). We searched for articles, chapters, and reviews using key terms in the title: “orange peel*”, “orange pomace”, “orange by-product*”, “orange byproduct*”, and “orange waste*”. About 80% of the publications were from the last decade. Therefore, we repeated the search queries, limiting them to the past 10 years (2013–2023) and to articles in English. We exported the dataset to VOSviewer© software (version 1.6.14) for keyword co-occurrence analysis. We set the following parameters: a minimum of 5 occurrences, association strength for normalization, a cluster resolution of 1.00 with merged small clusters, and optimized labels for file export. Furthermore, we processed the available responses for duplication and relevance, and then manually screened the articles used for this scoping review, categorizing them by theme. The main steps of this bibliometric workflow are summarized in Figure 2, which illustrates the sequential procedure followed for data collection, refinement, export, and analysis.

### 2.2. Overall Data Analysis

**Figure 3:**
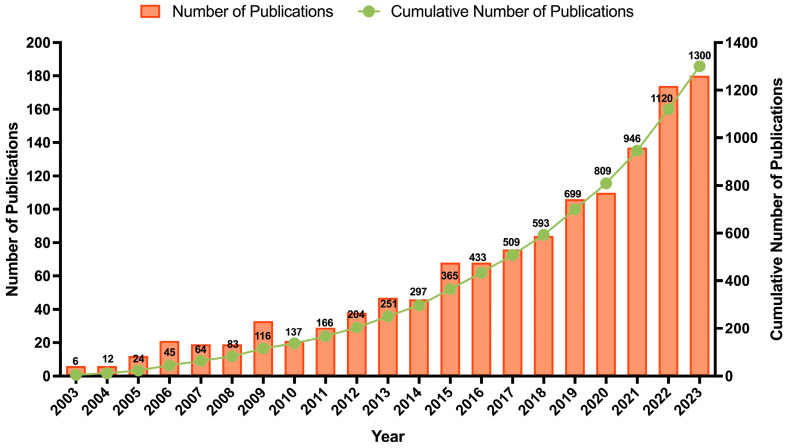
Trend analysis of annual publications on orange peels, waste, pomace, or by-products in the last twenty years (2003–2023).

From 2003 until 2023, a total of 1300 documents were published on this topic (1264 articles, 21 book chapters, and 15 reviews). The evolution of the research activity over this period is presented in Figure 3. An interesting conclusion is that over the last decade, the number of published documents (80.69%; n = 1049) was significantly higher than that of the previous decade (19.31%; n = 251), demonstrating the increased interest and investment in the upcycling and valorization theme. The authors observed that half of the documents focused on three subject areas: agricultural and biological sciences (14.7%), chemistry (13.8%), and environmental science (11.6%). Many studies have explored the extraction of BCs, such as pectin and antioxidant extracts, while recent documents have assessed their bioactive effects and applications. Within the agricultural and biological sciences area, 47% of publications are categorized under food sciences. Notably, over 85% of these publications were produced in the last decade, with prominent contributions from journals such as *Food Chemistry* (n = 16), *Journal of Food Processing and Preservation* (n = 14), and *LWT—food science and technology* (n = 13). This shift might be attributed to the growing global focus on sustainability and circular economy practices, advancements in extraction technologies, increased consumer demand for natural and functional products, and stricter policies promoting food waste valorization.

Additionally, 15 chapters and 21 reviews focusing on OP were published over the past two decades. Among the book chapters, 52% addressed the development of novel adsorbent solutions for the textile and water treatment industries, while 43% concentrated on the upcycling of OP to create new ingredients for various applications. Across the reviewed literature, a growing consensus exists on the versatile potential of OP as a source of BCs for high-value applications. Several book chapters converge on this point, emphasizing its use in food, cosmetics, and nutraceutical formulations. Interestingly, the functional use of OP flour in meat products, as shown by Pérez-Chabela et al. [20], exemplifies the ingredient’s technological adaptability beyond conventional categories. However, most contributions remain fragmented, focusing either on single BCs or isolated applications. To address this, we recently proposed an integrated OP upcycling strategy that targets the sequential recovery of EOs, hesperidin-rich polyphenols, and pectin, laying the groundwork for a scalable biorefinery model aligned with green chemistry and circular economy principles [13].

Regarding reviews, 21% focused on EO extraction, while 35% discussed advances in using OP to produce value-added bioproducts mainly for food, feed, nutraceuticals, and organic fertilizer applications. Only a small portion of reviews highlighted the upcycling of OP from a biorefinery perspective. For instance, Rezzadori et al. [21] proposed various strategies to enhance OP value, including the production of feed ingredients, EO, and pectin, as well as the production of ethanol, biogas, and limonene through integrated processes. Their work featured detailed flowcharts, mass balances, economic and environmental assessments, and investment requirements. More recent narrative reviews have summarized the best green extraction methods used to obtain upcycled ingredients and their applications in functional foods across various sectors, including baked goods, dairy, meat, and beverages [12,22,23]. Despite significant contributions to understanding OP as a valuable functional food ingredient, a notable gap remains in the literature regarding the nutraceutical potential of OP for therapeutic and prophylactic treatment in humans, particularly for the prevention and treatment of NCDs. This gap underscores the need for further research to investigate the application of OP-derived ingredients as functional health-promoting agents, highlighting their therapeutic and prophylactic potential in the context of chronic disease prevention. However, a recent systematic review investigated the biological effects of OP on metabolic biomarkers, with the results showing that OP positively influenced lipid and glucose profiles, particularly in animal models [24]. While this systematic review provides critical insights into the biological effects of OP on metabolic biomarkers, our review builds upon these findings by exploring sustainable upcycling methodologies, innovative extraction techniques, and broader implications for gastrointestinal and cardiometabolic health.

**Figure 4:**
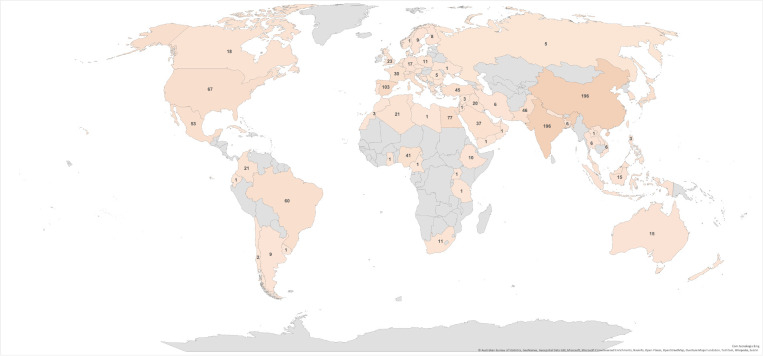
Geographical distribution of total published articles from 2003 to 2023.

In addition to the number and topics of publications, it is interesting to examine the geographic distribution of the topics (Figure 4). The number of publications by geographic location revealed that China had the most publications on the subject (15.6%), followed by India (15.1%) and Spain (7.9%). However, grouping the Mediterranean countries reaches a higher publication percentage (28.7%) than China, India, and Brazil. These countries produce large quantities of citrus fruits, particularly oranges, resulting in significant by-product volumes. Consequently, there is a burgeoning research trend in these nations focused on finding new and innovative methods to valorize this waste, which subsequently drives up the publication rate.

**Figure 5:**
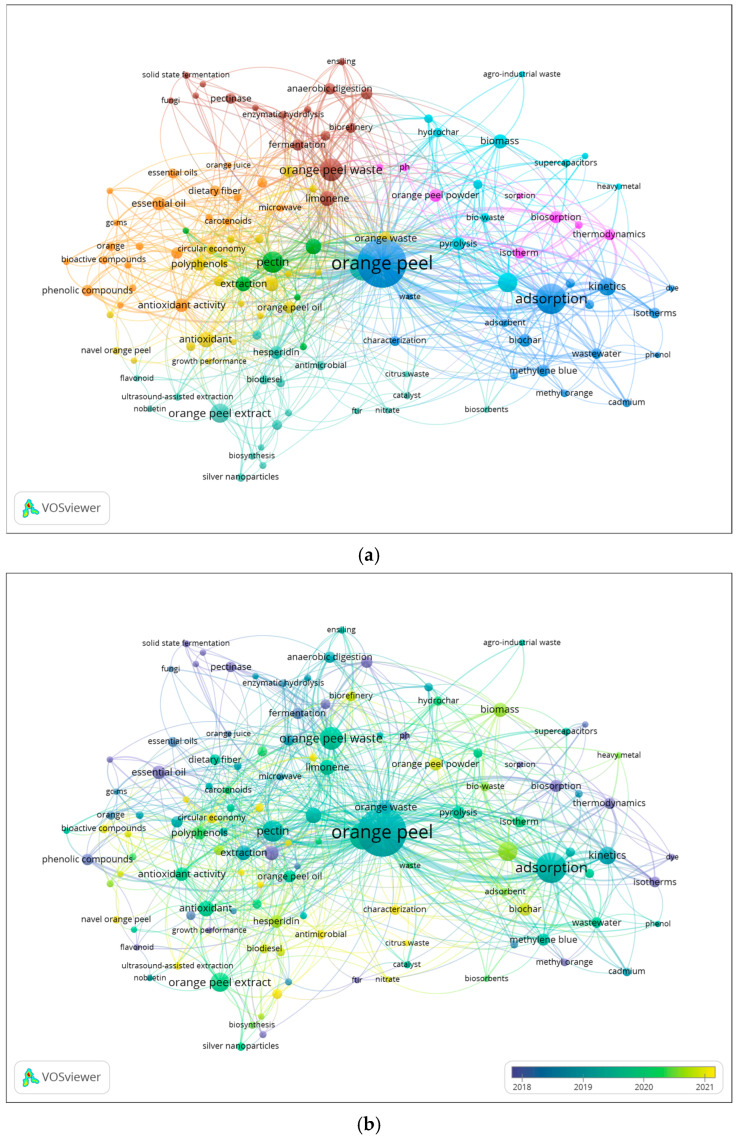
Network map of co-occurrence of author keywords divided into clusters (**a**) and divided by publication data (**b**) based on Scopus data (articles in English published between 2013 and 2023).

The total number of research articles from the last ten years was analyzed in Vosviewer^®^ to ascertain the co-occurrence of author keywords. The bibliometric map (Figure 5a) illustrates a network of keywords, highlighting thematic relationships among research topics. Node size is proportional to the number of publications associated with each keyword. The nodes are grouped into clusters, each represented by a color. Additionally, the distance between keywords reflects the strength of their association, with shorter distances indicating stronger associations within the research topics. A total of 2583 keyword co-occurrences were identified across the publications, with at least five keywords appearing together in the title, abstract, or keyword list. As a result, 124 keywords were grouped into eight distinct clusters. The network of keywords is centered on OP, which appeared in 204 studies, followed by other representative keywords such as adsorption, kinetics, activated carbon, and extraction. The keywords in dark and light blue, pink, and brown clusters are related to environmental studies. Interestingly, the brown cluster is correlated with the biorefinery concept, which connects with the green, orange, and yellow clusters. These clusters focus on key BCs extracted from OP, such as hesperidin, pectin, polyphenol-rich extracts, and EOs, highlighting their diverse applications in upcycled ingredients for food and nutraceuticals. In order to further understand how these research topics have evolved over time, a second analysis was generated. Figure 5b presents the temporal evolution of keywords, where the yellow nodes correspond to the recent research keywords that emerged mainly from 2021 onwards. This analysis reveals a clear shift in OP research over the last decade. Earlier studies (blue nodes) focused mainly on wastewater treatment, heavy metal removal, and adsorption, reflecting the traditional environmental engineering perspective on OP as a low-cost adsorbent. In contrast, the most recent keywords (green to yellow color gradient) such as biorefinery, circular economy, green extraction, polyphenols, and pectin highlight a transition toward high-value upcycled ingredients and sustainable food waste valorization.

The appearance of terms like antioxidant, antimicrobial, and dietary fiber further indicates a transition toward health-related applications, consistent with current consumer and regulatory priorities. Collectively, these trends demonstrate a clear transition in scientific interest, from the earlier focus on environmental remediation and low-value waste treatment toward OP upcycling for the development of high-value functional ingredients. This evolution underscores the scientific and industrial shift toward sustainable innovation and aligns with global priorities for a circular bioeconomy and sustainability.

Despite this growing focus, a notable gap exists in the literature regarding the health and nutraceutical benefits of upcycled OP ingredients. While keywords such as “antioxidant” and “antimicrobial” indicate some investigation into these attributes, there is a marked absence of research into other health benefits, such as anti-inflammatory effects and gut modulation, as well as a lack of human clinical trials. This gap hinders the ability to translate the bioactive properties observed in vitro into practical, real-world health interventions. To address these limitations, there is an urgent need for clinical studies to evaluate the efficacy of OP-derived products in disease prevention and health promotion. Additionally, while various extraction techniques, including ultrasound-assisted (UAE) and microwave-assisted (MAE) extraction, are well established, there remains an underexplored opportunity to enhance extraction yields and quality through more advanced methodologies such as supercritical CO_2_ and enzyme-assisted extraction. Optimizing these advanced techniques aligns with broader objectives of green chemistry principles, offering significant potential to advance the field of natural products, particularly in the areas of health and nutrition.

**Figure 6:**
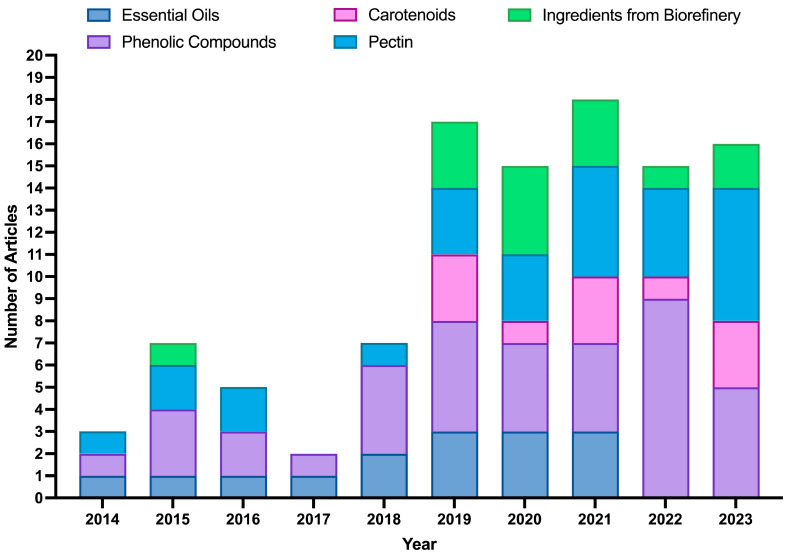
Annual distribution (2014–2023) of scientific articles focused on the extraction of ingredients from orange peels. The publications were categorized according to the main targeted ingredients extracted: essential oils, phenolic compounds, carotenoids, pectin, and ingredients obtained through integrated biorefinery approaches.

With the same metadata, research articles were manually separated into several categories relevant to the scoping review on the area of extraction. Figure 6 shows articles related to the extraction of BCs (pectin, EOs, polyphenol-rich extracts (such as hesperidin extracts), and carotenoids) as well as articles on extraction based on the biorefinery concept. The data show a clear upward trend in the number of articles published from 2018 onwards, which aligns with the growing global interest in the upcycling of by-products. This rise can be attributed to the increasing recognition of OP and other by-products as valuable sources of BCs with applications in food, nutraceuticals, and pharmaceuticals.

Overall, phenolic compounds (42%) and pectin (29%) emerged as the most studied categories, reflecting their broad applications in nutraceuticals, particularly for gut health. Publications on phenolic compounds and pectin extraction have shown a steady increase, peaking in 2022 and 2023. In contrast, EOs and carotenoids accounted for fewer studies (about 17 and 22%, respectively), although their growing interest follows the global demand for natural colorants and flavor ingredients. Regarding extraction technologies, hydrodistillation (HD) and MAE were the most applied for EOs (together accounting for 62% of the studies). In comparison, solid–liquid extraction (SLE) (25%) and UAE (23%) were the leading methods for phenolic compounds. Regarding pectin, SLE with a hot acid medium solvent was the method most cited in the literature (48%).

Beyond these single-compound extraction methods, research has also started to explore more integrated approaches. Biorefineries represent a newer and emerging area, as extracting multiple BCs from a single raw material offers significant economic benefits by diversifying the revenue streams. This approach is consistent with global shifts toward sustainable production and circular economy principles, aiming to maximize value from by-products while minimizing waste, in line with initiatives such as the European Green Deal and the Zero Waste concept. Since 2019, the biorefinery concept has gained increasing attention in OP upcycling. These strategies maximize biomass utilization and improve overall profitability, while generating co-products such as nutraceuticals, sorbents for water treatment, and bio-combustibles, demonstrated at different scales (industrial, pilot, and laboratory). In this context, 14 research studies have already proposed OP valorization through integrated biorefinery processes, designed to obtain different value-added ingredients and energy vectors. More than 50% of these studies focused on the recovery of ingredients for food and nutraceutical applications, mainly EOs, polyphenol-rich extracts, and pectin. Altogether, these findings highlight the growing interest in holistic valorization models that move beyond conventional single-compound recovery and accelerate the transition towards a sustainable circular bioeconomy.

## 3. Nutritional and Phytochemical Compounds in Orange Peels

**Figure 7:**
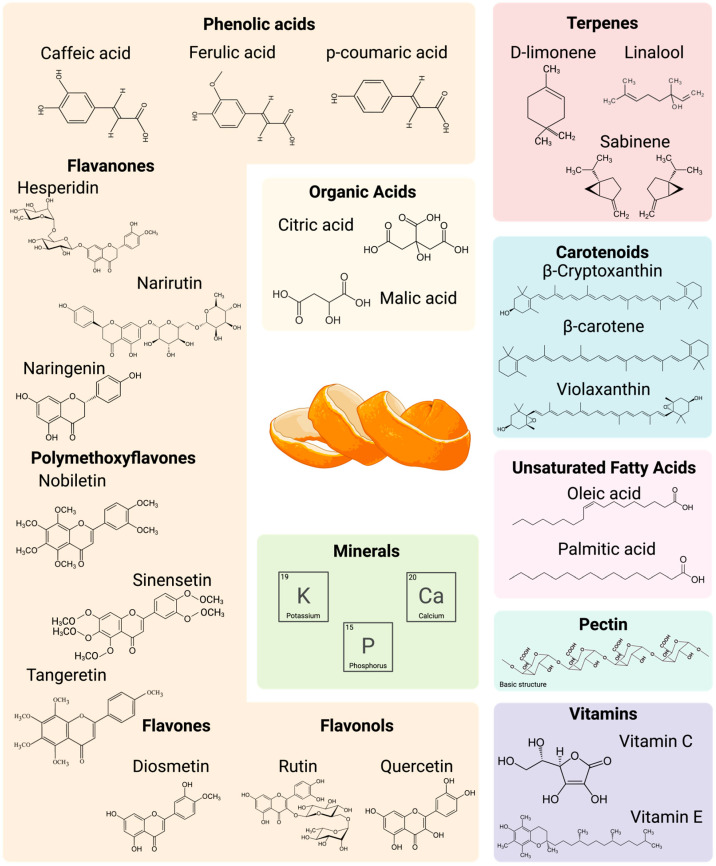
Spectrum of most representative nutrients and phytochemicals present in orange peels.

The species *Citrus sinensis* (L.) Osbeck, commonly known as the sweet orange, includes various cultivars, such as Navel and Valencia, and pigmented varieties like Moro and Tarocco [25]. Due to the genetic diversity among these cultivars, the nutritional and phytochemical composition of their peels can vary significantly. OP contains various compounds, including fiber, protein, minerals, phenolic compounds, volatiles, and fats, which are distributed across two main layers. The albedo (mesocarp), the white middle layer attached to the pulp, primarily comprises pectin and hemicellulose, forming a net-like structure. The outer layer, known as the flavedo (epicarp), is the colored surface of the orange, where sebaceous glands are concentrated. This layer contains terpenes, fats, volatile compounds, carotenoids, and phenolic compounds [26]. An illustration of the most relevant nutrients and phytochemicals, along with a detailed summary of the proximate and structural composition of OP from various varieties, is provided in Figure 7 and Table 1, respectively. These values are crucial for selecting the most effective upcycling pathway since they are affected by various biotic and abiotic factors, including cultivar, geographic location, and processing methods. In this context, the compositional variability observed across studies has important practical implications for both extraction and storage processes.

Moisture content emerges as a critical, yet often underappreciated, factor in the valorization of OP. Reported values vary widely from 6.48% to 81.6% (Table 1), reflecting differences in sample state (fresh vs. dried), processing protocols, and reporting units, which hinders cross-study comparability. For instance, M’hiri et al. [27] report 81.6% in fresh material, while other studies report much lower values, likely reflecting dry weight data. Beyond comparability, moisture directly shapes operations: high moisture accelerates enzymatic and microbial activity, shortens shelf-life, dilutes target solutes, and impairs solvent penetration, often necessitating rapid dehydration (e.g., convective or freeze-drying) and cold storage; low-moisture matrices improve mass transfer, shorten extraction time, and reduce thermal/energy load during processing. Moisture also influences solvent choice (e.g., hydroalcoholic vs. non-polar systems) and process design (solid–liquid ratio, residence time, temperature). Despite this impact, moisture normalization is rarely implemented; standardizing to a dry basis, reporting water activity and drying pretreatments, and correcting yields/purities to DW would substantially improve methodological rigor and enable more reliable scale-up in biorefinery workflows.

The lipid content of OP ranges from 0.08% to 7.90%, indicating that beyond EOs, OP is a minor yet relevant source of bioactive lipids, particularly unsaturated fatty acids (UFAs) [28]. Although the total lipid fraction is modest, the UFAs profile is enriched in oleic (C18:1) and linoleic (C18:2) acids, with lower amounts of linolenic acid (C18:3), alongside saturated fractions such as palmitic acid (C16:0) concentrated mainly in the flavedo waxes. These four fatty acids make up over 90% of all fatty acids in OP [29]. Composition varies with cultivar, fruit part (flavedo vs. albedo vs. pulps), and maturity, and can be altered by drying/storage because UFAs are susceptible to oxidation [30]. Nevertheless, to the best of our knowledge, OP has not been systematically evaluated as a primary source of UFAs, as its lipid yield is comparatively low when contrasted with other agri-food side streams (e.g., seed oils or microalgal biomass). Fatty acids may co-extract with other lipophilic compounds such as EOs and carotenoids (see Section 3.2). In carotenoid workflows, residual lipids can aid solubilization and subsequent bioaccessibility, but should be accompanied by appropriate purification steps. The high content of lipids, mainly EOs, can form emulsions that hinder hydroalcoholic extraction; therefore, EO removal as the first step of the integrated extraction process improves selectivity and yield.

In terms of protein content, OP exhibits a broader range, from 1.6% to 8.12%, suggesting that certain varieties of OP may be particularly rich in protein, potentially making them a valuable source for protein extraction. The presence of significant protein levels also opens avenues for developing plant-based protein supplements, and further research is needed to understand the specific amino acid profiles and their nutritional relevance. Additionally, enzymes such pectinesterase are already known to be present in higher concentrations in OP [31]. Other important micronutrients are reported in the literature to be present in OP, including minerals (potassium and phosphorus) [31] and vitamins (C, E, complex B, and A) [28,32].

The fiber content of OP is especially noteworthy, with total fiber levels up to 65.40%. Insoluble fiber (approximately 70%) is linked to improved bowel regularity and cholesterol management via increased bile acid excretion [33,34,35], whereas soluble fiber (approximately 30%) helps modulate post-prandial blood glucose and supports the gut microbiota through fermentable substrates that yield short-chain fatty acids (SCFAs) [18,35]. Mechanistically, insoluble fiber increases fecal bulk and reduces transit time while lowering LDL cholesterol via enhanced fecal bile–acid losses, which stimulate hepatic bile–acid synthesis and LDL receptor-mediated clearance [35,36]. In contrast, soluble fibers such as pectin form viscous gels that attenuate post-prandial glucose absorption and are fermented to SCFAs that support gut barrier function and endocrine signaling (GLP-1/PYY) while modulating hepatic cholesterol synthesis [34].

The balance between insoluble/soluble fiber is cultivar-dependent and influenced by raw material storage; endogenous enzymes can weaken the fiber network if no pretreatments are applied, which matters when selecting OP sources aligned with specific health/processing targets. Structural carbohydrates, such as cellulose, hemicellulose, and lignin, are crucial for both biomass conversion and bioethanol production. The high cellulose content (up to 60.96%) suggests that OP could be an effective raw material for biofuel production, aligning with the growing emphasis on sustainable and renewable energy sources [37]. Importantly, this compositional variability also alters processing behavior since a higher insoluble fiber content increases matrix rigidity and tortuosity, reducing solvent accessibility and mass transfer; in practice, cell disruption pretreatments like milling or UAE with a higher solid–liquid ratio ensure wettability [31,38].

The presence of pectin (ranging from 6.15% to 18.6%) further supports potential extraction from OP, as it is widely used in the food, nutraceutical, and pharmaceutical industries [39,40,41]. The variability in pectin across studies indicates that certain varieties may be better suited for industrial pectin extraction, while abiotic factors also affect pectin levels in OP. Pectinesterase and other carbohydrases can reduce pectin during storage/extraction, degrading gelling capacity. This enzymatic activity helps explain the DE variability reported across studies and the outcomes. In contrast to insoluble fiber, soluble fiber/pectin-rich matrices swell in hydroalcoholic media, which can facilitate the diffusion of mid-polarity flavanones and enable efficient pectin recovery; however, the viscous swollen phase can entrap non-polar carotenoids [42,43], justifying prior EO removal/defatting and/or the use of co-solvents in carotenoid workflows.

The differences in total carbohydrate composition and sugar content across studies also suggest that OP may serve as an alternative feedstock for natural sweeteners or low-calorie food ingredients [26,44]. For instance, the presence of total sugars ranging from 18.60% to 46.24% highlights OP as a potential candidate for sugar production or fermentation processes [44]. In addition, OP contains a relevant pool of organic acids, with citric acid dominating, followed by malic, ascorbic, and succinic acids. Their relative abundance varies with cultivar, fruit part (flavedo/albedo/endocarp), and maturity, and can be further affected by processing and drying conditions. Functionally, these acids lower matrix pH and can chelate metals, which favors acid-mediated pectin extraction and helps limit pectinesterase activity during storage. In biorefinery schemes, endogenous organic acids can also support fermentative routes [45], and notably, citric acid can be extracted from OP and valorized as a natural preservative for clean-label products [46]. A recent study by Fernandes et al. [46] recovered 7 g citric acid per 100 g dry OP using UAE with water as a solvent under low-power/longer time conditions. From a processing standpoint, high sugar and organic acid levels lower the glass transition temperature and promote stickiness and browning during drying, increasing losses of phenolic compounds and carotenoids; therefore, low-temperature dehydration or freeze-drying techniques, followed by tight moisture/aw control, are recommended to preserve the stability of BCs and improve downstream green extraction efficiency. Nutritional analysis of OP faces challenges due to research scope, OP variability, and inconsistent methods and units used in experimental research. This inconsistency makes it difficult to compare existing results. Additionally, many studies fail to report key parameters such as lipids, minerals, organic acids, peptides, or enzyme composition. The lack of standardized methodologies, including unit homogenization and comprehensive data collection, further complicates the interpretation and application of OP composition data. This review highlights the importance of consistent data collection and transparent reporting within both scientific and industrial communities. Standardizing research methodologies and improving the accessibility of information will facilitate more informed decision-making regarding the valorization of OP and its diverse applications.

Secondary metabolites, including phenolic compounds, Eos, and carotenoids, are synthesized in OP. Recent reviews have extensively covered citrus by-products, comparing orange, lemon, mandarin, and grapefruit [16,26,33,36]. However, a dedicated compendium focused solely on OP’s secondary metabolites is needed.

**Table 1:** Ten-year overview of proximate and structural composition of orange peels from various cultivars and under different study scopes.

Year	2015	2017	2018	2019	2021	2022	2023	2019	2024	2024
Purpose/Scope	NA	F	PE	NAA	BE	NAA	DF	BE	PE	POL
**Moisture**	76.02 *	81.60 *	-	40.00 *	78.53 *	9.18	6.48	-	11.76	
**Ash**	3.17	4.91	4.30	4.30	3.61	3.00	3.83	3.70	0.09	3.00
**Fat**	0.80	-	2.75	7.90	5.18	3.52		-	5.35	
**Protein**	8.12	1.60	5.26	2.83	4.86	6.72	6.04	-		4.90
**Total Fiber**	10.00	-	9.90	4.65	44.91	13.30	65.40	-		46.50
**Insoluble Fiber**	-	-	59.19	-	-	-	50.92	-		37.30
**Soluble Fiber**	-	-	18.60	-	-	-	14.48	-	50.45	9.20
**Total Carbohydrates**	-	-	77.79	20.32	-	33.55	84.34	-	70.60	45.60
**Total Sugars**	46.24	-	18.60	-	-	-	-	35.2	-	-
**Structural Carbohydrates**										
**Cellulose**	17.52	12.70	-	69.09	30.17	-	25.87	18.6	-	-
**Hemicellulose**	-	5.30	-	5.43	9.35	-	14.21	14.3	-	-
**Lignin**	14.38	0.20	-	19.80	5.07	-	8.77	6.5	-	-
**Pectin**	15.72	-	-	-	11.18	-	12.60	18.6	18.18	-
**Reference**	[47]	[48]	[49]	[44,50]	[37]	[51]	[42]	[52]	[53]	[35]

-: non reported parameters; *: fresh weight (% FW); parameters without an asterisk are reported in dry weight (% DW). Abbreviations: NA—natural antioxidants; F—fermentation; PE—pectin extraction; NAA—natural antioxidants and antimicrobials; BE—biorefinery extraction; DF—dietary fiber, POL—polyphenols.

### 3.1. Essential Oils

EOs account for 3–4% of OP dry weight (DW) and represent a complex mixture of aromatic compounds, mainly volatile compounds (80–99%). Their composition is shaped by multiple factors, including cultivar, fruit maturity, edaphoclimatic conditions, and extraction methods [54,55]. While several reviews have characterized EO profiles across citrus species [56,57], notable inconsistencies persist, even within *Citrus sinensis* L., due to variability in genetic and environmental factors. This compositional heterogeneity poses a challenge for standardization and reproducibility in both research and industrial applications. Despite the well-documented dominance of limonene and related monoterpenes, further comparative studies are needed to clarify the extent to which these variables modulate EO quality particularly when aiming for consistent bioactivity or regulatory compliance in food, cosmetic, or nutraceutical formulations.

The volatile fraction is composed mainly of 80% hydrocarbon monoterpenes (limonene, β-pinene, α-pinene, myrcene, and sabinene), 10% oxygenated monoterpenes (linalool, α-terpineol), 2% hydrocarbon sesquiterpenes (valencene and β-caryophyllene), and 2% oxygenated sesquiterpenes (β-Sinensal) [58,59,60]. The non-volatile fraction mainly comprises long-chain hydrocarbons, sterols, wax, and limonoids. Overall, it is difficult to quantitatively compare the results between studies because concentrations are expressed in different units, such as % *w*/*w* or peak area (%). Some compounds consistently appear to be the most abundant. Limonene is typically the most abundant component, with peak area percentages ranging from 73.9% to 98% across different studies [60,61,62]. Other compounds, such as β-pinene, ⍺-pinene, myrcene, sabinene, and linalool, are also frequently detected at relatively higher concentrations. The composition of EOs significantly impacts both the flavor and the bioactive properties. The EOs from OP are Generally Recognized as Safe (GRAS), allowing for extensive applications across the cosmetic, food, and pharmaceutical sectors due to their antimicrobial, anti-inflammatory, and antioxidant activity, primarily stemming from their high limonene content [63].

A notable gap in the literature is the variability in non-volatile compounds that could be attributed to the different extraction methods applied to obtain this fraction, and this will be explored in the next section. Further research is needed to standardize extraction techniques and consider environmental and storage factors influencing EO quality, ensuring comparability across different research studies.

### 3.2. Carotenoids

Carotenoids are a ubiquitous class of isoprenoid-pigmented phytochemicals involved in photosynthesis and signaling in OP, contributing to their vibrant color and significant health benefits. During fruit maturation, carotenoid content increases while chlorophyll is degraded due to the gradual conversion of chloroplasts into chromoplasts [64]. Based on their chemical structure, carotenes are classified into two main groups: (i) carotenes—linear or cyclic hydrocarbon chains (e.g., α- and β-carotene, lycopene; and (ii) xanthophylls—oxygenated derivatives of carotenes (e.g., violaxanthin, lutein, β-cryptoxanthin) [65]. Xanthophylls are found in free form or as fatty acid esters because their oxygenated functional groups can bind with fatty acids. On the other hand, carotenes, which have a simple hydrocarbon structure without oxygenated functional groups, are only found in free form. OP is a good source of xanthophylls, mainly acylated with saturated and unsaturated fatty acids; however, it depends mainly on the variety of oranges, the growing conditions, and the maturity of the fruit. For instance, at the fully mature stage, the total carotenoid contents of the flavedo of sweet orange were nine-fold higher (12.6 mg/100 g FW) than those in the pulp (1.4 mg/100 g FW) [64]. In this study, the most abundant carotenoids in the endocarp were violaxanthin, monoesters, and diesters containing acyl groups like laurate and oleate. The other major carotenoids were lutein and β-carotene. In contrast, in this study, violaxanthin, lutein, α-carotene, and β-carotene were also found to be prevalent in the flavedo of fully mature green fruits. The β-carotene content in OP has been widely studied and is considered one of its most significant carotenoids, with concentrations typically ranging from 1.0% to 2.0% of the dry weight, depending on the cultivar and environmental factors. β-cryptoxanthin is another key carotenoid in OP, with levels typically around 0.2% to 0.5% of dry weight.

Beyond their role as photosynthetic pigments, carotenoids are industrially relevant because provitamin A species (β-carotene, β-cryptoxanthin) and xanthophylls (lutein/zeaxanthin) contribute to nutritional and antioxidant functionality, which supports their use as clean-label colorants and as actives in nutraceutical/functional food and cosmetic formulations [16]. In addition, carotenoids from orange are permitted for use as pigments in feed additives [22].

### 3.3. Phenolic Compounds

OP is a rich source of phenolic compounds, particularly flavonoids such as flavanones, flavones, flavonols, and phenolic acids [66]. These secondary metabolites are known for their bioactive properties and contribute to the prevention of various diseases. On average, OP contains 534 mg GAE/100 g dry weight (DW) of phenolic compounds, with varying concentrations and composition throughout the fruit’s development, maturation, and across cultivars. OP is especially rich in flavones like hesperidin, naringin, and narirutin, and polyethoxylated flavones (PMFs) such as nobiletin, sinensetin, and tangeretin [66]. Unlike the juice and pulp, OP is abundant in less polar flavonoids and flavone aglycones, along with PMFs, which are less soluble than other flavonoid forms. Hesperidin stands out for its high concentration, ranging from 80 to 400 mg per 100 g DW. Narirutin is another significant flavonoid, with typical concentrations between 50 and 150 mg per 100 g DW, while naringin contributes less but still plays a significant role in the phenolic profile of OP, reaching up to 50 mg per 100 g DW. Although present in smaller amounts, phenolic acids are also important contributors to the bioactivity of OP. The major phenolic acids are from the hydroxycinnamic group, including ferulic, caffeic, chlorogenic, and p-coumaric acid. Ferulic acid is the most abundant, with concentrations ranging from 5 to 25 mg per 100 g DW, while caffeic and p-coumaric acid are typically found in lower amounts (2 to 10 mg per 100 g DW). For instance, Ozturk et al. [7] highlighted that ferulic acid predominates in OP, with lower levels of p-coumaric acid, typically concentrated in the albedo. Interestingly, phenolic compounds in OP are often bound to the cell wall and predominantly associated with fibers. This property underscores the importance of optimizing extraction methods to release phenolic compounds from OP efficiently.

The high diversity and concentration of secondary metabolites highlight OP’s potential as a natural source of extracts, particularly valuable for the nutraceutical industry in disease prevention and treatment. By harnessing these compounds, industries can add significant nutritional and functional value to their products while promoting sustainable practices and reducing environmental impact. However, despite extensive research on polyphenols, considerable variability persists in the concentration of these compounds, which are influenced mainly by natural conditions. This variability presents challenges in standardizing OP-derived products. For instance, Ozturk et al. [7] reported geographical differences in the phenolic profile of OP. Oranges collected from China contained higher levels of PMFs (nobiletin and tangeretin) and exhibited greater antioxidant capacity compared to those from the USA.

## 4. Extraction of Upcycled Ingredients from Orange Peels

The recovery of BCs using eco-friendly methodologies stands at the forefront of agro-food research. Extraction plays a vital role in obtaining BCs from by-products. Various extraction methods can be employed, but the primary goal is to maximize both yield and quality. This must be achieved while minimizing time, energy consumption, solvent use, environmental impact, economic costs, and waste [14]. Nevertheless, achieving high-purity BCs derived from food by-products for human consumption remains challenging. Recent advancements in green extraction methods, spurred by the Twelve Principles of Green Chemistry [67], emphasize minimizing energy consumption, utilizing sustainable solvents and renewable natural products, and ensuring the safety and high quality of the extracts/ingredients. While numerous reviews have explored both conventional and green extraction approaches for citrus by-products, most address citrus waste broadly and offer limited specificity for OP. Collectively, the literature highlights a growing preference for green extraction methods such as MAE, EAE, UAE, PFE, and SFE, due to their ability to improve extract purity, reduce solvent use, and enhance process efficiency. However, these studies rarely compare methods side-by-side in the context of OP or consider their integration into scalable biorefinery systems. This lack of consolidation hinders the identification of optimal strategies tailored to OP’s matrix and compound diversity. By addressing this gap, the present review aims to critically map and compare current extraction technologies applied specifically to OP valorization under a sustainability and zero-waste lens.

The most reported green methods often face high equipment costs and challenges in industrial scale-up. Conventional methods, such as SLE, remain widely industrially used, but green principles provide guidance for its improved sustainability and safety in product extraction. The suitable choice of solvent method, along with operating parameters including time, temperature, and the solid–liquid ratio, significantly affects the composition and bioactive properties of the extract. However, what is interesting about both conventional and green extraction methods is their versatility. They can extract different BCs by adjusting the solvent and operating parameters. This section reviews the latest advancements in green extraction for the valorization of OP and the extraction of its key BCs.

### 4.1. Essential Oil Extraction

The European Pharmacopeia allows for HD and cold pressing to extract citrus EOs [68]. Cold pressing has gained popularity in recent years due to its higher yield and lower energy consumption compared to HD. This method involves mechanically pressing the peel to rupture oil glands, followed by centrifugation to separate the EOs [69]. However, cold-pressed EOs may contain phototoxic compounds, leading to skin irritation. This limitation has prompted the development of green extraction methods which aim to reduce environmental impact and improve efficiency. UAE [70], UAE combined with enzymatic hydrolysis [71], steam explosion followed by HD [62], and MAE [72] have been proposed as promising alternatives. Table 2 summarizes recent studies on EO extraction. It is possible to conclude that HD and MAE are the most applied extraction techniques in the literature (62%). The yield and chemical composition of EOs vary considerably depending on the extraction method. For example, HD can yield up to 1.9% EOs, with limonene concentrations ranging from 78 to 96%. Steam treatment releases EO droplets, which are then separated as the vapor condenses [69]. However, limitations such as high energy consumption and long extraction times remain significant barriers. Green extraction methods such as UAE, MAE, and steam explosion are designed to minimize extraction time while maximizing yields. The main advantages of MAE are time reductions and solvent-free extraction, known as solvent-free MAE [58,73]. For instance, microwave hydrodiffusion and gravity (MHG) has been successfully applied for solvent-free EO extraction. A study by Bustamante et al. [59] achieved an optimal yield of 4.22% in just 15 min, while traditional HD took 240 min to yield 4.16%. However, a notable gap in the current literature is the lack of large-scale commercial applications for some of the green extraction methods, such as MHG [59], despite it showing promise as a candidate for industrial scale-up for the large-scale extraction of EOs. As European legislation mandates that EOs from citrus be extracted using HD or cold pressing, HD remains the preferred method for industrial extraction, despite the growing shift towards greener alternatives. These findings could encourage legislative changes to support the transition to more sustainable extraction techniques. Additionally, further research is needed to address the phototoxic compounds found in cold-pressed EO. Standardizing extraction conditions, such as temperature, solvent choice, and extraction time, is essential to ensure consistency and reliability in EO yields and chemical composition across studies.

### 4.2. Phenolic Compound Extraction

An increasing number of studies on the extraction of phenolic compounds from OP highlight their health-promoting properties and potential industrial applications. Table 3 summarizes the most recent studies in this field. Recent studies show a strong trend toward green extraction methods, with SLE and UAE being the most widely used (48% of studies). UAE has been shown to provide higher yields compared to SLE, due to the cavitation process that disrupts the plant matrix [27,74]. SLE consumes less energy than UAE, reducing the overall energy footprint of the extraction process. It is a conventional extraction method used for extracting phenolic compounds from by-products, utilizing food-grade solvents such as ethanol or hydroethanolic mixtures [75,76]. SLE is a simple and cost-effective extraction technique that does not require advanced equipment and is widely used in industrial processes without significant capex investment. Additionally, solvent recovery enhances its environmental friendliness. By optimizing extraction parameters and using green solvents, SLE becomes an environmentally friendly and safe alternative for extract phenolic compounds from OP, particularly benefiting small- to medium-sized enterprises. It has the potential to significantly enhance the citrus processing industry and serve as a model for producing cleaner, high-demand phenolic compounds. In contrast, UAE involves high-frequency sound waves to generate high temperature and pressure through bubble cavitation [77], which facilitates extraction. Similar advantages are observed by [27,74], reporting a hesperidin concentration 1.5-fold higher compared to SLE. MAE was set for 3 and 6 min, respectively, while SLE and UAE required 30 min. MAE uses electromagnetic waves to interact with polar solvents like water and ethanol. This interaction causes rapid matrix disruption through uniform heating, resulting in shorter extraction times and lower energy consumption. In addition, this technology could be improved by using fresh material, therefore, in situ raw material water works as solvent. Positive results are reported for obtaining extracts rich in phenolic compounds from OP using MHG [78]; however, further research is needed.

Supercritical fluid extraction (SFE) and pressurized fluid extraction (PFE), which account for 15% of studies, also show promising results. SFE uses CO_2_ and PEF solvents like ethanol, water, or methanol under high pressure. While SFE yields higher results than SLE, it is less efficient than MAE and UAE for extracting hesperidin [27]. Works from Leo et al. [79] using pressurized water showed lower concentrations of hesperidin (35.70 mg/g DE) when using PEF compared to UAE (278.95 mg/g DE). Conversely, Barrales et al. [80] found PEF using ethanol 50% (*v*/*v*) to yield 8 times more hesperidin and a higher narirutin concentration. The variations between studies are mainly due to the choice of solvent, as methanol and ethanol generally yield better results than water due to the polarity of flavonoids. The lack of standardized extraction parameters makes it difficult to compare results, highlighting the need for consistent solvents and conditions to accurately assess the impact of extraction technologies.

Based on the data, 9% of the studies on phenolic compounds extracted from OP use DESs or ionic liquids (ILs). They are a greener, GRAS-approved alternative to ethanol that are safe for direct human consumption. Studies highlight choline chloride [7] and lactic acid or glucose [81]. However, time and the solid–liquid ratio affect yield and composition, so optimizing conditions through the design of experiments is recommended. Other methods recently tested include EAE [82], hydrodynamic cavitation [83], and combined methods such as ultrasonic-assisted enzymatic processes [84]. SLE and UAE remain the most recognized and effective methods for OP upcycling for polyphenol-rich extract development. To optimize yield while minimizing both time and energy consumption, the implementation of pretreatments, extraction combinations, and simultaneous extractions is highly recommended.

### 4.3. Carotenoid Extraction

Along with phenolic compounds, carotenoids are important BCs found in OP. However, studies on carotenoid extraction from OP remain limited, with recent methods focusing on natural deep eutectic solvents (DESs) or ionic liquids (ILs) as solvents (Table 4). Despite growing interest in carotenoid extraction from OP, many studies still prioritize total yield extraction over detailed compositional analysis. A prevalent limitation in the literature is the omission of carotenoid profiling, even when using different extraction methodologies such as UAE and DESs, with several works reporting extraction efficiency while neglecting to characterize the specific compounds recovered [85,86,87,88]. This lack of data hinders comparative assessments and the development of application-specific extracts. Methodologically, carotenoid recovery from OP remains challenging due to the rigid cell wall structure of OP and the strong binding of carotenoids to macromolecules such as fatty acids. Pretreatments like drying and grinding are frequently employed to address these barriers. Recent findings suggest that the presence of in situ water can significantly reduce extraction efficiency, reinforcing the need for prior dehydration through freeze-drying or convective drying to enhance solvent penetration and the release of lipophilic compounds [89,90]. Technologies such as UAE, MAE, hydrodynamic cavitation, PEF, or high-pressure extraction could disrupt intramolecular forces and facilitate solvent penetration, thus increasing extraction yield without dehydrating the raw material.

The combination of UAE and D-limonene shows promise as a green extraction method for obtaining carotenoids from OP, yielding 11.25 mg/L and increasing carotenoid content by 40% compared to conventional extraction [91]. On the other hand, SFE with CO_2_ offers higher purity and yield compared to other methods and eliminates harmful solvent residues. Usually, SFE provides lower yields of polar carotenoids (e.g., xanthophylls), although this can be improved by adding ethanol as a co-solvent. DESs and ILs show significant potential for carotenoid extraction, particularly due to their eco-friendly properties. However, they remain underexplored, particularly in integrated processes where solvents with recyclability potential could extract BCs from waste sources, ultimately contributing to a circular economy. Although DESs and ILs are routinely described as scalable and suitable for large-scale extraction, robust industrial scale-up and economic viability are still insufficiently demonstrated. In practice, the high viscosity limits mass transfer (frequently demanding water or process intensification such as UAE/MAE/PEF). Several preparation routes are energy- and time-intensive, and product/solvent recovery and recycling can add downstream unit operations (e.g., resin-based recovery) that increase the cost at scale. For food-grade applications, a fuller assessment of safety/toxicity and chemical stability of DES systems is still needed. In contrast, ILs appear mainly in comparative studies; cost and toxicological/regulatory concerns make them less attractive than DESs for the extraction of carotenoids intended for foods, which explains the current emphasis on DESs as the preferred “greener” option [92].

The variability in carotenoid yields and purity across different methods highlights the importance of standardized protocols to improve reproducibility and scalability.

### 4.4. Pectin Extraction

Currently, most commercially available pectin is obtained by upcycling citrus peels. The conventional industrial method, SLE using an acid-mediated solvent and heating (hot acid extraction), is preferred due to its high yield, simplicity, and cost-effectiveness [93]. Organic acids like citric acid are now used in place of inorganic acids, offering similar or even better pectin yields while causing less degradation of the pectin structure, which enhances its bioactive properties. This approach aligns with green chemistry principles and supports the production of clean-label food ingredients. Pectin extraction from OP has been extensively studied, showing varying results in yield and composition (Table 5). Nearly 48% of studies utilize acid-mediated hot extraction, with recent research exploring sustainable alternatives to replace inorganic acids. Overall, extraction factors include particle size, raw material moisture content, pH, temperature, extraction time, and solvent type, all of which significantly affect pectin yield. Most studies aim to optimize these parameters, typically reporting an ideal temperature range of 80–95 °C for efficient extraction. While higher temperatures can enhance yield by disrupting cell walls, they can also lead to decreased yield due to excessive depolymerization beyond certain limits.

MAE is a promising green technology that heats the solvent more quickly than acid-mediated hot extraction, yielding pectin with a higher galacturonic acid (GalA) content. For example, Zioga et al. [39] found that 3 minutes of MAE could achieve comparable yields to 117 min of HCl-mediated extraction. Citric acid-mediated extraction for 106 min yielded slightly more pectin (22%) but without a significant statistical difference. In addition, MAE can produce food-grade pectin (GalA content > 65%) with a degree of esterification (DE) at 74%. The FAO has established that food-grade pectin must contain more than 65% GalA. This high DE indicates minimal chain de-esterification during extraction. Studies from Kute et al. [40] reported similar findings, suggesting that prolonged extraction periods at high temperatures might lower DE values. Although UAE has shown potential, no significant reduction in extraction time was observed compared to traditional processes. High energy requirements and specialized equipment currently limit its industrial scalability. There is a gap in research examining different extraction durations with UAE to determine if higher yields could be achieved in less time. Optimizing UAE could reduce energy consumption and improve efficiency, making it more suitable for large-scale pectin production. However, it is possible to conclude that UAE can decrease the optimal temperature used in acid-mediated hot extraction to achieve the maximum pectin yield. In this context, studies from Bosch et al. [41] demonstrated that the combination of UAE with enzymatic treatment resulted in a higher yield (27%) compared to HCl-mediated hot extraction (22%), although the process required a longer extraction time.. Nonetheless, the lower molecular weight and DE of the UAE-extracted pectin allow it to serve as a food additive where higher solubility and gelling properties are desired, mainly for low-sugar products. Furthermore, pectin is increasingly recognized for its health benefits stemming from specific structural domains with bioactive properties. Thus, the extraction method significantly influences pectin’s potential as a nutraceutical [94].

Innovative methods such as ohmic heating (Saberian et al. [95]), induced electric fields [96], and pulsed extraction [31] have shown positive yields. However, limited pectin characterization makes their advantages unclear. While no studies have focused specifically on EAE during the last decade, it could yield high pectin, as evidenced by its successful application to other by-products. EAE offers benefits over traditional methods, such as enhanced yields by breaking down plant cell walls at lower temperatures, which saves energy. Moreover, EAE does not need an extra step for waste neutralization. However, one of the main drawbacks of EAE is the high cost of enzymes. While green technologies like MAE and UAE reduce energy consumption and extraction time, there is still a need to standardize extraction conditions. Additionally, most of these green methods are still in the research phase, and further optimization is required before they can be implemented on a larger industrial scale. MAE is superior in reducing extraction time and increasing pectin yield compared to conventional acidic extraction, although scaling it up for industrial use is challenging. Currently, acid-mediated hot extraction using organic acids is the most affordable green extraction method.

**Table 2:** Experimental conditions, yield, and chemical composition of EOs extracted from orange peels through various extraction methodologies.

By-Product Type	Extraction	Extraction Conditions			Yield (%)	Identification and Quantification (%)	Ref.
		Time (min)	Solvent	Other Parameters			
Fresh OP	Enzyme-assisted HD	260	Water	3.9% Viscozyme L^®^; 55 °C	4.6	Limonene (66) and β-caryophyllene (23)	[97]
	HD	35		Electromagnetic induction heating method	3.77	Limonene (94.43), β-myrcene (2.16), sabinene (0.47), linalool (0.29), valencene (0.17)	[61]
		41		Heating mantle	2.72	Limonene (93.2), β-myrcene (2.28), sabinene (0.77), linalool (0.63%), valencene (0.04)	
	HD assisted by solar energy	120		10 m^2^ solar reflector coupled to a HD unit	1.03	Limonene (95.96), sabinene (0.16), myrcene (1.70), α-pinene (0.37), linalool (0.23)	[60]
	HD	190		-	1.05	Limonene (95.24), sabinene (0.19), myrcene (1.73), α-pinene (0.39), linalool (0.30)	
	MAE	60		EthosX Extractor (600 W; 100 °C)	0.43	Limonene (80)	[72]
Fresh OP (Valencia variety)	Solvent-free MAE	30	-	1000 W; 100 °C	0.40	Limonene (94.64), sabinene (0.54), myrcene (1.64), α-pinene (0.43), linalool (0.62)	[73]
	HD	180	Water	-	0.40	Limonene (95.48), sabinene (0.49), myrcene (1.87), α-pinene (0.53), linalool (0.30)	
	CP	-		Automated cold-pressing machine	0.16	Limonene (95.06), sabinene (0.54), myrcene (1.82), α-pinene (0.51), linalool (0.30)	
	Steam explosion + HD	30		Pretreatment: 240 s, 170 °C, 8 bar	1.34	Limonene (89.13), myrcene (3.41), nonane (2.00), dodecanal (0.82), α-pinene (0.72)	[62]
	HD	240		-	1.21	Limonene (77.39), myrcene (6.08), linalool (5.13), decanal (2.92), octanol (2.18)	
Fresh OP (Navel Navelate variety)	Microwave-assisted HD	20		785 W for 5 min + 250 W for 15 min	1.80	Limonene (97.38), β-myrcene (0.79), sabinene (0.50) and α-pinene (0.39)	[59]
	HD	240		-	1.70	Limonene (96.75), β-myrcene (0.74), sabinene (0.49) and α-pinene (0.32)	
Fresh Orange Flavedo	Salt-assisted extraction by HD	210	Water + NaCl	1:8.4 SLR; NaCl 5.3% (*m*/*v*); petroleum ether as used for EO separation	2.15	Limonene (88.07), β-myrcene (4.93), α-pinene (1.14), sabinene (0.39), α-citral (0.36), linalool (0.26),	[98]
Fresh OP (after juice extraction)	HD	180	Water	-	1.90	Limonene (86.70), sabinene (2.90), β-pinene (3.10), linalool (2.40), α-terpineol (1.91)	[58]
	Salt-assisted extraction by hydrodistillation	180	Water + CaCl_2_	-	3.0	Limonene (87.90), sabinene (2.18), β-pinene (2.95), linalool (3.05), α-terpineol (2.01)	
	EAE	240	Water	mix pectinase/hemicellulases; 60 min; 40 °C	3.7	Limonene (88.3), sabinene (2.28), β-pinene (3.01), linalool (2.61), α-terpineol (1.83)	
	UAE + HD	210		30 min; 25 °C with power of 700 W	2.9	Limonene (88.2), sabinene (2.34), β-pinene (3.05), linalool (2.5), α-terpineol (1.72)	
	Solvent-free MAE	30	-	10 min soaked with water; Power of 500 W	3.6	Limonene (85.4), sabinene (1.8), β-pinene (2.75), linalool (4.8), α-terpineol (2.50)	

HD—hydrodistillation; CP—cold pressing; MAE—microwave-assisted extraction; UAE—ultrasound-assisted extraction; EAE—enzyme-assisted extraction.

**Table 3:** Experimental conditions, yield, and chemical composition of extracts rich in phenolic compounds extracted from orange peels through various extraction methodologies.

By-Product Type	Extraction	Extraction Conditions			Identification and Quantification (mg/g DW)	Ref.
		Time (min)	Solvent/Medium	Other Parameters		
**Dried OP Powder**	SLE	30	Ethanol 80% (*v*/*v*)	1:10 SLR; orbital shaker 900 rpm; 99.85 °C	Hesperidin (34.9), ferulic acid (0.10), catechin (0.06), rutin (0.04)	[74]
	UAE	30		1:10 SLR; power of 35 W; 99.85 °C	Hesperidin (40.0), ferulic acid (0.05), rutin (0.03), catechin (0.02)	
	MAE	6		1:10 SLR; stirring at 1000 rpm; 99.85 °C	Hesperidin (58.2), catechin (0.15), ferulic acid (0.125)	
	SLE	30		1:10 SLR; 35 °C	Neohesperidin (5.51), hesperidin (8.6), narirutin (0.38), nobiletin (0.42), naringin (0.42)	[27]
	UAE	30		1:10 SLR; 125 W; 30 °C	Neohesperidin (9.86), hesperidin (8.36), narirutin (0.17), nobiletin (0.74), naringin (0.82)	
	MAE	3		1:10 SLR; power: 200 W, 76 °C	Nehohesperidin (12.2), hesperidin (9.3), naringin (1.3), nobiletin (0.8), didymin (0.6)	
	SFE	30	CO_2_	1:10 SLR; 250 bar, 10 MPa, flow rate of 15 g/min, 80 °C	Neohesperidin (7.0), hesperidin (5.1), naringin (0.65), nobiletin (0.5), didymin (0.24)	
**Freeze-dried OP**	SLE	36	Methanol/DMSO (1:4 *v*/*v*)	1:20 SLR; rotary shaker, 150 rpm	Hesperidin (0.23), sinensetin (0.39), nobiletin (0.37), luteolin (0.04), rutin (0.03), tangeretin (0.02)	[82]
	EAE	240	pectinase, cellulase, hemicellulose, and papain	1:20 SLR; rotary shaker, 150 rpm	Hesperidin (0.29), sinensetin (0.56), nobiletin (0.49), luteolin (0.08), rutin (0.16), tangeretin (0.03)	
	UAE	27	Methanol/DMSO (1:4 *v*/*v*)	1:17.57 SLR; ultrasonic power, 63.84 W; 25.70 °C	Hesperidin (0.23), sinensetin (0.6), nobiletin (0.44), luteolin (0.08), rutin (0.04), tangeretin (0.03)	
	PFE	50	Water	60 °C	Hesperidin (35.7), narirutin (0.90), gallic acid (0.21), p-coumaric acid (0.11), ferulic acid (0.1) (in DE)	[79]
	UAE	10	Methanol	1:33 SLR; sonicator water bath; 40 °C	Hesperidin (278.95), narirutin (0.14), gallic acid (0.23), p-coumaric acid (0.11), ferulic acid (0.39) (in DE)	
**Dried OP**	SLE	-	Water	1:10 SLR; 25 °C	Narirutin (194), naringin (157.2), hesperidin (158.6)	[63]
		-	Ethanol		Narirutin (198.6), naringin: (182.1), hesperetin (117.9), naringenin (1.2)	
	DES	100	choline chloride-Ethylene Glycol ([Ch]Cl:EG 1:4)	1:10 SLR; temperature of 99.85 °C	Gallic acid, syringic acid, rutin, naringin, p-coumaric acid, ferulic acid, caffeic acid, trans-cinnamic acid, flavone, and thymol	[7]
**OP** **(after the processing of juice and EO)**	PFE	40	Ethanol 50% (*v*/*v*)	1:5 SLR; 65 °C	Hesperidin (58), naringin (0.4), narirutin (9), hesperitin (0.27), tangeritin (0.32), naringenin (0.48) (in DE)	[80]
	UAE	30	Ethanol 50% (*v*/*v*)	1:56.5 SLR; 15 min in ultrasonic bath at 30 °C and 15 min in shaker at 200 rpm	Hesperidin (7.10), gallic acid (0.15), narirutin (5.50), hesperitin (0.24), tangeritin (0.26), naringenin (0.25) (in DE)	
	SFE	240	CO_2_	28.7 MPa; 60 °C	Hesperidin (0.11), caffeic acid (0.6), luteolin-6-C-glucoside (0.13), myricetin (0.38), apigenin-7-O-rutinoside (0.26)	[99]
**Defatted orange peels**	SFE	10	water	1:24 SLR; temperature: 150 °C; 10 MPa	Hesperidin (20), narirutin (2.33)	[100]
	UAE	120	Ethanol 80% (*v*/*v*)	1:20 SLR; 35 °C; 800 W power and 20 kHz frequency	Hesperidin (3), narirutin (2.85)	
	SLE			1:20 SLR; 35 °C; 200 rpm shaker	Hesperidin (1.56), narirutin (0.73)	

-: non-defined. Abbreviations: OP—orange peel; SLE—solid–liquid extraction; UAE—ultrasound-assisted extraction; MAE—microwave-assisted extraction; EAE—enzyme-assisted extraction; PFE—pressurized fluid-assisted extraction; SFE—supercritical-assisted extraction; SLR—solid–liquid ratio; DE—dry extract.

**Table 4:** Experimental conditions, yield, and major carotenoids extracted from orange peels through various extraction technologies.

By-Product	Extraction Technologies	Extraction Conditions			Yield (%)	Identification and Quantification (mg/g DW)	Refs.
		Time (min)	Solvent	Other Parameters			
**Freeze-dried OP**	SLE	-	Acetone	Without saponification	-	Free carotenoids (50.9), monoesters (29.3), diesters (20.3), total carotenoids (97.4)	[101,102]
	UAE + IL	30	[C4mim]Cl	1:3 SLR; ultrasound probe at 20 kHz and 200 W, at 80% amplitude	-	Free carotenoids (32.1), monoesters (24.6), diesters (7.6), total carotenoids (64.2)	
		25	[C4mim]Cl (ethanol 59% (*v*/*v*) as co-solvent)	1:3 SLR; ultrasound probe at 160 W, in an ice bath for 5 min; Amberlite XAD-7HP resin was used to separate the carotenoids from the IL	-	(all-E)-lutein (10.1), (all-E)-β-carotene (6.48), (13Z)-violaxanthin-C12: (3.54)	
	SFE	16	Supercritical CO_2_ and methanol	Temperature of 80 °C; 150 bar	-	free carotenoids (lutein, zeaxanthin, β-cryptoxanthin, b-carotene), carotenoid esters (antheraxanthin-C12:0, zeaxanthin-C12:0, β-cryptoxanthin-C12:0), apocarotenoids (β-apo-8′-carotenal, apo-8-luteinal), apo-esters (apo-10′-zeaxanthinal-C4:0, apo-8′-zeaxanthinal-C6:0)	
**Freeze-dried OP (after juice extraction)**	UAE + IL	5	[BMIM][Cl] (ethanol 50% (*v*/*v*) as co-solvent)	1:3 SLR; ultrasound probe at 200 W and 20 kHz at 80% amplitude; Amberlite XAD-7HP resin was used to separate the carotenoids from the IL	0.32	Total carotenoids (17.95), 9-cis-violaxanthin (6.76), all-trans-violaxanthin (0.48), all-trans-lutein (2.87)	[103]
	SLE	1440	Acetone	Saponified with 10% methanolic KOH overnight at room temperature	0.78	Total carotenoids (7.88), 9-cis-violaxanthin (1.53), all-trans-violaxanthin, all-trans-lutein (3.07)	
**Dried OP** **(Navel cultivar)**	DES + UAE	20	Octanoic acid: Proline	1:20 SLR; ultrasound intensity of 60% (120 W); 45 °C	0.46	-	[104]
	SLE		Hexane		0.39	-	

-: non-defined. Abbreviations: OP—orange peel; SLR, solid-to-liquid ratio; UAE—ultrasound-assisted extraction; IL—ionic liquid; DES—deep eutectic solvent.

**Table 5:** Experimental conditions, yield, and characteristics of pectin extracted from orange peels through various extraction technologies.

By-Product	Extraction Technologies	Extraction Conditions						Yield (%)	Degree of Esterification (DE) (%)	Other Pectin Parameters	Ref.
		Time (min)	Temperature (°C)	pH	Solvent/Medium	Purification Method	Other Parameters				
**Dried OP**	SLE (hot acid-assisted extraction)	240	80	1.5	Water with HCl	Ethanol cold precipitation; oven drying	1:20 SLR	22.1	55	GalA: 20.91%; Mw: 197.78 kDa;	[41]
	UAE + enzymes	270	80 °C (30 min) and 50 °C (240 min)	5.0	50 mM sodium citrate buffer with Celluclast		1:19 SLR; 30 min in ultrasonic bath (300 W); then incubated at 70 rpm	26.9	8	GalA: 22.77%; Mw: 70.22 kDa;	
	Ohmic extraction	1	90	1.5	Water with HCl	-	1:20 SLR; voltage: 30 V/cm	10.36	75	GalA: 68.24%	[95]
**Freeze-dried OP powder**	Acid-mediated hot extraction	60	90	2.0	Water with HCl	Nanofiltration (200 Da dialysis bag; ethanol precipitation; freeze-drying)	1:30 SLR	20.8	72	Mw: 212.9 kDa	[38]
	Hydrothermal Extraction	45	120		Water			18.9	64	Mw: 109.2 kDa	
**Dried OP Powder**	SLE (hot acid-assisted extraction)	114	94	1.45	Water with HCl	Ethanol cold precipitation; oven drying	1:20 SLR; water bath extraction	23.64	73	Anhydrouronic acid: 38.60%	[105]
	MAE	1.5	80	1.5	Water with HNO_3_	Ethanol cold precipitation; freeze-drying	1:20 SLR; frequency 2450 MHz and power 540 w	15.79	42	-	[40]
	SLE (hot acid-assisted extraction)	10			Water with HNO_3_		1:20 SLR	8.78	36	-	
**Dried OP powder** **(after juice processing; sugars and phenolics recovery)**	MAE	3	-	1.5	Water acidified with hydrochloric acid	Ethanol cold precipitation; oven drying	1:25 SLR; household microwave oven at 2450 MHz and irradiation 620 W	18.3	74	GalA: 65.7%; intrinsic viscosity: 0.57 dL/g; Mw: 9.9 kDa	[39]
	SLE (hot acid-assisted extraction)	117	90	1.6	Water with HCl		1:30 SLR	18.5	78	GalA: 56.4%; intrinsic viscosity: 0.774 dL/g; Mw: 14.6 kDa	
		160		2.0	Water with citric acid			22.8	56	GalA: 46.7%; intrinsic viscosity: 0.397 dL/g; Mw: 6.2 kDa	

-: non-defined. Abbreviations: OP—orange peel; SLR, solid-to-liquid ratio; SLE—solvent extraction; UAE—ultrasound-assisted extraction; MAE—microwave-assisted extraction; Mw—molecular weight; GalA—galacturonic acid.

**Figure 8:**
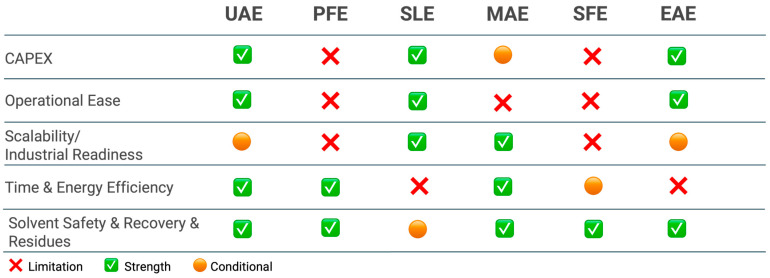
Comparative assessment of extraction methods for upcycled ingredients from orange peels. Abbreviations: UAE—ultrasound-assisted extraction; MAE—microwave-assisted extraction; PFE—pulsed-assisted extraction; SLE—solid-liquid extraction; SFE—supercritical fluid extraction; EAE—Enzyme-assisted extraction.

To complement the extraction section, Figure 8 provides a qualitative, side-by-side assessment of the main extraction techniques in terms of CAPEX, operational ease, industrial readiness, time/energy efficiency, and solvent safety/recovery.

As the Figure 8 indicates, conventional SLE remains the workhorse at scale because it combines low equipment costs, simple operation, and high throughput. Its main drawbacks are long extraction times, high solvent consumption, and a greater burden on solvent recovery/waste handling; nonetheless, when EtOH–H_2_O is used, safety is compatible with food-grade requirements and the economics are often favorable, explaining its persistent prevalence in industry. Among intensified “green” methods, UAE and MAE show clear strengths in time and energy efficiency and often preserve compound integrity. UAE is versatile for polyphenols and as an intensification step for pectin; at a large scale, performance can be equipment-dependent (cavitation distribution), which is why Figure 8 marks industrial readiness as conditional. MAE achieves one-order-of-magnitude shorter cycle times and competitive yields, but requires thermal control to avoid degradation of heat-sensitive targets (e.g., some carotenoids).

Pressurized liquid extraction (PFE) balances short extraction times with moderate CAPEX. Using EtOH–H_2_O under pressure gives good recoveries of phenolic compounds (and can aid pectin) with relatively low solvent footprints; the trade-off is pressurized operation, which raises training and safety requirements. SFE with CO_2_ excels in solvent safety and residue-free products and provides high purity/selectivity for carotenoids and EOs (co-solvent ethanol widens polarity). Its limitations are higher CAPEX and compressor energy, hence the conditional mark for time/energy and scalability.

Finally, EAE is attractive under mild conditions and with aqueous media, particularly for pectin and selected phenolics, aligning well with safety and “clean-label” expectations. The current bottlenecks are enzyme cost, longer residence times, and the need for downstream clarification, so industrial readiness is use-dependent (often best as a pretreatment rather than a stand-alone unit).

## 5. Increasing the Value of OP Through Biorefinery Processes

As previously reported, the nutritional and phytochemical composition of OP enables the generation of valuable sub-fractions by applying different green extraction methods. However, the first steps towards waste valorization and circular economy implementation focus on recovering a single BC, as discussed previously, leading to suboptimal use of biomass and increased waste. Since OP contains many high-value-added BCs (such as EO, pectin, and polyphenols), using a biorefinery approach has the advantage of producing multiple ingredients and maximizing the value derived from the biomass feedstock while producing a lower amount of waste to be treated. A biorefinery approach is defined as “the sustainable processing of biomass into a spectrum of marketable products” by the International Energy Agency (IEA). Therefore, it is a compendium of integrated processes that selectively target the extraction of value-added ingredients, preserving the integrity of the biomass for subsequent extractions. This approach offers a more integrated extraction method aligned with green chemistry principle 4, the Sustainable Development Goals, and, more importantly, the new European Union strategies such as Farm-to-Fork that seek to prevent food losses and waste, promote a circular economy, and ensure the maximum reuse of materials. Green technologies and solvents can provide efficient extraction with low solvent and energy consumption in the biorefinery system. Indeed, several valorization pathways under the biorefinery concept have been proposed; nevertheless, most extraction and bio-based products lack a high technological readiness level (TRL), inhibiting their introduction as a feasible option at the industrial level. Table 6 highlights key biorefinery strategies designed to valorize OP, including feasibility analyses and cost estimations.

Over 60% of these studies target the recovery of food-grade compounds such as EOs, polyphenols (mainly hesperidin), pectin, and sugarsg Generally extracted using either conventional or green physical processes. The complexity of biorefineries varies, ranging from simple set-ups extracting two ingredients to more advanced systems that produce energy. Notably, Figueira et al. [43] proposed a green method to co-extract pectin and hesperidin with high yields (18.7% and 1%, respectively), achieving efficient solvent reuse. In contrast, Jokic et al. [106] and Angoy et al. [107] implemented single-step green extractions using SFE, UAE, or MAE to recover EO and polyphenol-rich fractions. While these approaches are time-efficient, residual solid biomass often remains unutilized. This highlights a missed opportunity: applying a third extraction step could unlock pectin or energy recovery, thereby enhancing process circularity. Collectively, these cases reflect a shift toward more holistic OP valorization, though few fully exploit its multi-component potential. A three-step extraction strategy has been reported to sequentially recover EO, polyphenol-rich extracts, and pectin [60,108]. While both studies achieved high yields, only one adopted green extraction, reducing extraction time, energy use, and CO_2_ emissions. In contrast, steam distillation and citric acid-mediated hot extraction were associated with high energy demands. Although citric acid is less costly than inorganic acids, pectin extraction remains economically limiting. To address this, Tsouko et al. [108] proposed a process design that reduces energy requirements by 62% by concentrating the pectin-rich extract and recycling ethanol. Investment in green extraction, such as solar systems, incurs high initial costs and a long payback period. However, it offers an environmentally friendly solution compared to conventional systems that rely on energy from fossil sources. Some studies, in addition to physical processes, applied biotechnological or chemical processes to obtain biogas [37], activated carbon [109], or biofuel [110]. Integrated processes and solvent recycling have been found to reduce costs, payback time, and environmental impact. In most integrated biorefinery approaches, EOs are usually removed at the start of the process. While this may seem intuitive due to their high volatility and potential to interfere with downstream extraction steps, it is important to emphasize this rationale explicitly. Early EO removal not only prevents the degradation of thermolabile compounds but also aids in the subsequent recovery of more polar or thermally stable compounds.

Overall, most OP biorefinery papers prioritize polyphenol-rich extracts and pectin extraction (about 66% of the studies), mainly because of their economic value and high market demand. These compounds are highly valued in the food and pharmaceutical industries due to their versatility and known recognized benefits.

**Table 6:** Ten-year overview of biorefinery methodologies applied to fresh OP obtained after juice extraction.

Pretreatment	Extraction Technologies	Yield Ingredients Obtained (from 1 Ton of OP)	Main Findings on Feasibility Study and Cost Estimate	Ref.
Dried in an oven at 50 °C for 24 h and ground	Citric acid extraction Alkaline extraction Enzymatic hydrolysis	13 tons free sugars 2.9 tons pectin 0.8 tons XOS bioenergy	→Pectin and xylan precipitation are expensive. Therefore, 30% and 70% evaporation of pectin and xylan resulted in the lowest investment, payback time, ethanol, and energy consumption, respectively. →The integrated process reduced the net unit cost of pectin and XOS production. →The critical role of solvent and water recycling in reducing production costs and environmental footprint cannot be overstated. For instance, using citric acid not only improves economic feasibility but also significantly reduces the environmental impact, with 92% of the citric acid being recyclable. →XOS production is workable only if it is associated with the production of other bioproducts.	[111]
Cut to 1 cm and stored at 6 °C	Steam distillation Citric acid extraction Anaerobic digestion	6.07 kg EOs 22.72 kg pectin 77.71 Nm^3^ biogas	→The proposed OP biorefinery has high energy demands, with EO production using over 51% of the thermal energy and pectin extraction consuming about 36%. →The utility costs (44.8%) are the most representative of the process due to the high consumption of steam and cooling water in the EO and pectin production processes. →Capital depreciation costs are higher than the raw material costs. Therefore, applying this process at scales of <240 ton/day is not feasible without strategic planning and investment.	[37]
Dried at 50 °C and ground	Steam distillation UAE Citric acid-mediated hot extraction Enzymatic hydrolysis	1.5 kg EOs 1.3 kg polyphenol-rich extract 34 kg pectin 68 kg bacterial cellulose	→Industrial pectin and EO production are highly energy-consuming due to high utility requirements. →High quantities of ethanol used for pectin precipitation should be recycled. Process design showed that energy requirements could be reduced by 62% during pectin separation when the pectin-rich liquid extract is concentrated to 25% of its original volume via a mechanical vapor recompression-forced circulation evaporator system.	[108]
-	Solar HD HCl extraction	10 kg EOs 20 kg polyphenol-rich extract (78% hesperidin) 80 kg pectin	→The distillation time was reduced by more than 30% compared to the traditional method while also allowing for higher narirutin and hesperidin concentrations. →The conventional process emits about 2560 g of CO_2_, whereas solar HD has a zero CO_2_ footprint since it requires no additional energy. →The investment cost for a solar system is 28.5% higher than that for conventional processes, but it has a payback period of nearly two years with no additional expenses.	[60]
Blade-milled until 500 µm and frozen (−20 °C)	Acetone extraction Acid and enzymatic hydrolysis	22.5 kg EOs 15.7 kg polyphenol-rich extract 196.2 kg pectin 296 kg glucose	→Despite the purpose of sustainable integrated OP valorization, solvents such as acetone and petroleum ether were used. In addition, inorganic acid was used for pectin extraction. Monosaccharides were obtained solely based on green extraction (EAE).	[52]
Frozen and ground in ice (≤10 mm)	Hydrodynamic cavitation	6 g EO (96% limonene) 2.6 kg polyphenol-rich extract with hesperidin (5.68 mg/g) and naringin (2.56 mg/g) Pectin	→The process’s scalability was validated since 42 kg of OP was used to test the hydrodynamic cavitation equipment (semi-industrial scale). However, based on the energy balance, it would be imperative to optimize the extraction time. →The results presented in this study open the route to the integral valorization of OP via a simple, low-cost, and highly effective technology, requiring water as the unique additional raw material. →The overall specific energy consumed at the end was around 0.62 kWh/kg.	[83]

Abbreviations: OP—orange peel; EOs—essential oils; UAE—ultrasound-assisted extraction; XOS—xylooligosaccharides; EAE—enzyme-assisted extraction.

Therefore, the market price for these compounds is relatively high, providing a profitable return on investment. Briefly, the pectin global market is forecasted to reach about 103.990 tons and USD 1.85 billion in 2026, while in 2022, the global market size was estimated at USD 1.68 billion and is expected to grow at a compound annual growth rate (CAGR) of 7.4% from 2023 to 2030. This steady growth reflects the increasing consumer awareness of health benefits, the demand for natural products, and applications in multiple sectors such as dietary supplements and nutraceuticals. Last year, we proposed an integrated and sustainable protocol for OP valorization focusing on the recovery of BCs such as EOs, hesperidin-rich polyphenol extracts, and pectin [13].

Additionally, it is important to note that, to date, integrated processes specifically targeting carotenoids are largely lacking. Given their non-polar nature, future research should identify suitable extraction phases and integration points to enable their selective recovery within biorefinery frameworks.

The OP biorefinery, besides its great potential, faces challenges such as feedstock variability, seasonality, transportation, and process optimization, which can be overcome by focusing current and future research on improving process parameters, exploring alternative and green extraction methods, and integrating advanced biotechnological processes to enhance sustainability and reducing costs to increase profitability. Additionally, the processing unit for valorization should be close to processing facilities due to transportation challenges. In conclusion, the biorefinery demonstrates the practical application of green chemistry principles, SDGs, and EU farm-to-fork and green deal strategies. Converting waste into value-added ingredients addresses waste management issues, promotes environmental sustainability, and boosts economic growth. The diverse range of high-value ingredients offers new resources to support functional foods, nutraceuticals, and pharmaceutical industries. Continuous innovation and research in this area will be vital to support the EU’s objectives of transitioning to a circular economy and achieving climate neutrality by 2050.

**Figure 9:**
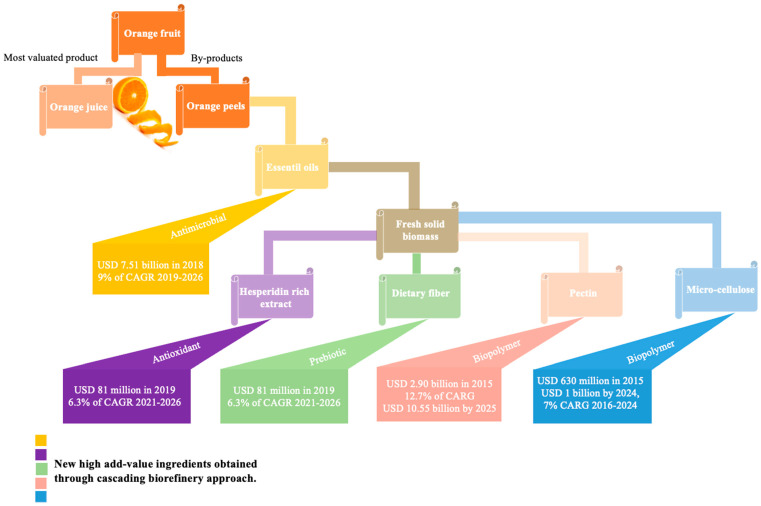
The cascade biorefinery approach applied to OP, illustrating the sequential recovery of high-value ingredients. Essential oils are first obtained, followed by the recovery of hesperidin-rich extracts, dietary fiber and/or pectin, and micro-cellulose until reaching the end of the biorefinery. Each stream is associated with specific bioactivities well described in the literature (antimicrobial, antioxidant, prebiotic, biopolymer) and market data, highlighting the economic potential of OP valorization within circular economy frameworks.

Figure 9 illustrates an example applied to the orange juice industry, where OP, traditionally considered a low-value residue, is converted into high value-added ingredients through different technological routes. This approach exemplifies how the principles of a circular economy can be integrated into food production systems, reducing waste and maximizing the value extracted from raw materials. One of the first steps in the cascade is the extraction of EOs, which are widely used in the food, cosmetic, and pharmaceutical industries due to their strong antimicrobial properties. This sector already represents a consolidated market, with a value of USD 7.51 billion in 2018 and an annual growth rate of 9% projected between 2019 and 2026. Beyond EOs, novel extraction and purification technologies enable the recovery of bioactive flavonoids such as hesperidin, a compound with demonstrated antioxidant and prebiotic functions. The hesperidin-rich extract market is still in its growth phase, but already accounted for USD 81 million in 2019, with an estimated CAGR of 6.3% until 2026, indicating increasing industrial interest. The remaining solid biomass can be further processed into dietary fiber, which contributes to gut health and offers applications as a functional food ingredient. In addition, polysaccharides such as pectin and micro-cellulose can be isolated as biopolymers, opening avenues for applications in food structuring, nutraceuticals, and bioplastics. The pectin market alone was valued at USD 2.90 billion in 2015 and is expected to surpass USD 10 billion by 2025, while micro-cellulose also shows significant growth projections.

Altogether, this cascading biorefinery model demonstrates the environmental, technological, and economic benefits of valorizing citrus side streams. By transforming residues into BCs and biopolymers, it provides a blueprint for sustainable innovation in the agri-food sector.

## 6. Conclusions and Future Perspectives

This review shows a clear shift from single-compound recovery to integrated, green extraction and biorefinery frameworks for OP, reflecting a global interest in circular models that minimize food waste and maximize the valorization of bioresources. Despite significant progress, the transition from laboratory-scale to industrial application remains limited, constrained by issues of scalability, process standardization, regulatory approval, and economic feasibility. Interest has grown markedly over the past decade, with polyphenol- and pectin-rich extracts emerging as the most studied and economically relevant upcycled ingredients. Their prominence in the literature reflects not only their natural abundance and well-documented health-promoting properties but also their strong compatibility with green extraction technologies. Polyphenols are known for their antioxidant and anti-inflammatory effects, while pectin is valued for its prebiotic activity and structural versatility as a functional ingredient. Their high economic value and market demand have made them central targets in most OP-based extraction strategies for food and nutraceutical applications. EOs are almost universally extracted at the beginning of integrated processes, due to their high volatility and potential interference with downstream operations, a strategy that, although widely applied, is rarely discussed in the literature as crucial to standardize biorefinery models. In contrast, carotenoids remain underexplored, with few efforts addressing their selective recovery. Their lipophilic nature poses specific extraction challenges, calling for innovative process integration strategies in future research.

Conventional extraction methods persist due to their simplicity and low capital requirements but are often time-, energy-, and solvent-intensive. Emerging green technologies such as, UAE, MAE, EAE, PFE, and SFE provide higher selectivity, shorter processing times, and reduced solvent use, yet high equipment costs and a lack of unified optimization parameters hinder their industrial implementation.

Future research should focus on (i) improving the technological readiness level of green extraction methods, (ii) enhancing integration and solvent recycling strategies, (iii) designing standardized protocols to allow for comparability between studies, and (iv) exploring new approaches to recover underexplored compounds such as carotenoids. Furthermore, interdisciplinary collaborations between academia and industry will be critical to overcoming economic and logistical barriers, ultimately enabling the large-scale application of OP integrative and biorefinery-based systems.

Such efforts will contribute to a more sustainable and health-oriented food system, aligned with the goals of the EU Green Deal and the UN Sustainable Development Goals. In addition, future work should not only address the optimization and upscaling of integrated biorefineries but also evaluate the functional and health-promoting potential of OP-derived ingredients. Since the main goal of upcycling is to translate these ingredients into food and nutraceutical applications, assessing their stability, bioaccessibility, bioactivity, and potential physiological benefits is essential. Bridging process optimization with biological validation will be key to unlocking the full prophylactic and/or therapeutic value of OP within a sustainable bioeconomy model.

## Data Availability

Data sharing is not applicable to this article as no new data were generated; all data analyzed in this study were compiled from previously published research accessed via the Scopus database. However, the data collection tables and summary files generated during the review process (such as bibliometric analyses and custom tables) are available from the corresponding author upon request.

## References

[1] Gavahian M., Chu Y., Mousavi Khaneghah A. (2019). Recent Advances in Orange Oil Extraction: An Opportunity for the Valorisation of Orange Peel Waste a Review. Int. J. Food Sci. Technol..

[2] Hou J., Liang L., Su M., Yang T., Mao X., Wang Y. (2021). Variations in Phenolic Acids and Antioxidant Activity of Navel Orange at Different Growth Stages. Food Chem..

[3] Statista Orange Production Worldwide. https://www.statista.com/statistics/577398/world-orange-production/.

[4] Duarte A., Fernandes M.J., Bernardes J.P., Miguel M.G. (2016). Citrus as a Component of the Mediterranean Diet. J. Spat. Organ. Dyn..

[5] Vlaicu P.A., Untea A.E., Panaite T.D., Turcu R.P. (2020). Effect of Dietary Orange and Grapefruit Peel on Growth Performance, Health Status, Meat Quality and Intestinal Microflora of Broiler Chickens. Ital. J. Anim. Sci..

[6] FAO (2021). Citrus Fruits Statistical Compendium.

[7] Ozturk B., Parkinson C., Gonzalez-Miquel M. (2018). Extraction of Polyphenolic Antioxidants from Orange Peel Waste Using Deep Eutectic Solvents. Sep. Purif. Technol..

[8] Panwar D., Saini A., Panesar P.S., Chopra H.K. (2021). Unraveling the Scientific Perspectives of Citrus By-Products Utilization: Progress towards Circular Economy. Trends Food Sci. Technol..

[9] Panwar D., Panesar P.S., Chopra H.K. (2021). Recent Trends on the Valorization Strategies for the Management of Citrus By-Products. Food Rev. Int..

[10] Xu G., Zhao J., Shi K., Xu Y., Hu H., Xu X., Hu T., Zhang P., Yao J., Pan S. (2023). Trends in Valorization of Citrus By-Products from the Net-Zero Perspective: Green Processing Innovation Combined with Applications in Emission Reduction. Trends Food Sci. Technol..

[11] Da Silva R.F., Carneiro C.N., de Sousa C.B.D.C., Gomez F.J.V., Espino M., Boiteux J., de los Á. Fernández M., Silva M.F., de S. Dias F. (2022). Sustainable Extraction Bioactive Compounds Procedures in Medicinal Plants Based on the Principles of Green Analytical Chemistry: A Review. Microchem. J..

[12] Mohsin A., Hussain M.H., Zaman W.Q., Mohsin M.Z., Zhang J., Liu Z., Tian X., Salim-ur-Rehman, Khan I.M., Niazi S. (2022). Advances in Sustainable Approaches Utilizing Orange Peel Waste to Produce Highly Value-Added Bioproducts. Crit. Rev. Biotechnol..

[13] Vilas-Boas A.A., Gómez-García R., Campos D.A., Correia M., Pintado M. (2023). Integrated Biorefinery Strategy for Orange Juice By-Products Valorization: A Sustainable Protocol to Obtain Bioactive Compounds. Food Waste Conversion.

[14] Chemat F., Abert-Vian M., Fabiano-Tixier A.S., Strube J., Uhlenbrock L., Gunjevic V., Cravotto G. (2019). Green Extraction of Natural Products. Origins, Current Status, and Future Challenges. TrAC Trends Anal. Chem..

[15] Burlea-Schiopoiu A., Ogarca R.F., Barbu C.M., Craciun L., Baloi I.C., Mihai L.S. (2021). The Impact of COVID-19 Pandemic on Food Waste Behaviour of Young People. J. Clean Prod..

[16] Saini R.K., Ranjit A., Sharma K., Prasad P., Shang X., Gowda K.G.M., Keum Y.-S. (2022). Bioactive Compounds of Citrus Fruits: A Review of Composition and Health Benefits of Carotenoids, Flavonoids, Limonoids, and Terpenes. Antioxidants.

[17] Wang M., Zhao H., Wen X., Ho C., Li S. (2021). Citrus Flavonoids and the Intestinal Barrier: Interactions and Effects. Compr. Rev. Food Sci. Food Saf..

[18] Guo Q., Hou X., Cui Q., Li S., Shen G., Luo Q., Wu H., Chen H., Liu Y., Chen A. (2024). Pectin Mediates the Mechanism of Host Blood Glucose Regulation through Intestinal Flora. Crit. Rev. Food Sci. Nutr..

[19] Turck D., Bohn T., Castenmiller J., De Henauw S., Hirsch-Ernst K.I., Maciuk A., Mangelsdorf I., McArdle H.J., Naska A., EFSA Panel on Nutrition, Novel Foods and Food Allergens (NDA) (2024). Safety of Glucosyl Hesperidin as a Novel Food Pursuant to Regulation (EU) 2015/2283. EFSA J..

[20] Pérez-Chabela M.L., Chaparro-Hernández J., Totosaus A. (2015). Dietary Fiber from Agroindustrial By-Products: Orange Peel Flour as Functional Ingredient in Meat Products. Dietary Fiber: Production Challenges, Food Sources and Health Benefits.

[21] Rezzadori K., Benedetti S., Amante E.R. (2012). Proposals for the Residues Recovery: Orange Waste as Raw Material for New Products. Food Bioprod. Process..

[22] Tahir Z., Khan M.I., Ashraf U., Adan I.R.D.N., Mubarik U. (2023). Industrial Application of Orange Peel Waste; a Review. Int. J. Agric. Biosci..

[23] Li Q., Putra N.R., Rizkiyah D.N., Abdul Aziz A.H., Irianto I., Qomariyah L. (2023). Orange Pomace and Peel Extraction Processes towards Sustainable Utilization: A Short Review. Molecules.

[24] Pineda-Lozano J.E., Fonseca-Bustos V., Martinez-Moreno A.G., Virgen-Carrillo C.A. (2022). The Biological Effect of Orange (*Citrus sinensis*, L.) by-Products on Metabolic Biomarkers: A Systematic Review. Front. Sustain. Food Syst..

[25] Seminara S., Bennici S., Di Guardo M., Caruso M., Gentile A., La Malfa S., Distefano G. (2023). Sweet Orange: Evolution, Characterization, Varieties, and Breeding Perspectives. Agriculture.

[26] Lu X., Zhao C., Shi H., Liao Y., Xu F., Du H., Xiao H., Zheng J. (2023). Nutrients and Bioactives in Citrus Fruits: Different Citrus Varieties, Fruit Parts, and Growth Stages. Crit. Rev. Food Sci. Nutr..

[27] M’hiri N., Ioannou I., Mihoubi Boudhrioua N., Ghoul M. (2015). Effect of Different Operating Conditions on the Extraction of Phenolic Compounds in Orange Peel. Food Bioprod. Process..

[28] Kotsou K., Chatzimitakos T., Athanasiadis V., Bozinou E., Adamaki-Sotiraki C., Rumbos C.I., Athanassiou C.G., Lalas S.I. (2023). Waste Orange Peels as a Feed Additive for the Enhancement of the Nutritional Value of Tenebrio Molitor. Foods.

[29] Jahromi K.G., Koochi Z.H., Kavoosi G., Shahsavar A. (2022). Manipulation of Fatty Acid Profile and Nutritional Quality of Chlorella Vulgaris by Supplementing with Citrus Peel Fatty Acid. Sci. Rep..

[30] Xie J., Cao Q., Wang W., Zhang H., Deng B. (2023). Understanding Changes in Volatile Compounds and Fatty Acids of Jincheng Orange Peel Oil at Different Growth Stages Using GC–MS. J. Integr. Agric..

[31] Patience N.A., Schieppati D., Boffito D.C. (2021). Continuous and Pulsed Ultrasound Pectin Extraction from Navel Orange Peels. Ultrason. Sonochem..

[32] Hassan F.A., Shalaby A.G., Elkassas N.E.M., El-Medany S.A., Hamdi Rabie A., Mahrose K., Abd El-Aziz A., Bassiony S. (2023). Efficacy of Ascorbic Acid and Different Sources of Orange Peel on Growth Performance, Gene Expression, Anti-Oxidant Status and Microbial Activity of Growing Rabbits under Hot Conditions. Anim. Biotechnol..

[33] Kaur S., Panesar P.S., Chopra H.K. (2023). Citrus Processing By-Products: An Overlooked Repository of Bioactive Compounds. Crit. Rev. Food Sci. Nutr..

[34] Bakr A.F., Farag M.A. (2023). Soluble Dietary Fibers as Antihyperlipidemic Agents: A Comprehensive Review to Maximize Their Health Benefits. ACS Omega.

[35] Núñez-Gómez V., Jesús Periago M., Luis Ordóñez-Díaz J., Pereira-Caro G., Manuel Moreno-Rojas J., González-Barrio R. (2024). Dietary Fibre Fractions Rich in (Poly)Phenols from Orange by-Products and Their Metabolisation by in Vitro Digestion and Colonic Fermentation. Food Res. Int..

[36] Andrade M.A., Barbosa C.H., Shah M.A., Ahmad N., Vilarinho F., Khwaldia K., Silva A.S., Ramos F. (2022). Citrus By-Products: Valuable Source of Bioactive Compounds for Food Applications. Antioxidants.

[37] Ortiz-Sanchez M., Solarte-Toro J.C., Orrego-Alzate C.E., Acosta-Medina C.D., Cardona-Alzate C.A. (2021). Integral Use of Orange Peel Waste through the Biorefinery Concept: An Experimental, Technical, Energy, and Economic Assessment. Biomass Convers. Biorefin..

[38] Wang T., Tao Y., Lai C., Huang C., Ling Z., Yong Q. (2023). Influence of Extraction Methods on Navel Orange Peel Pectin: Structural Characteristics, Antioxidant Activity and Cytoprotective Capacity. Int. J. Food Sci. Technol..

[39] Zioga M., Tsouko E., Maina S., Koutinas A., Mandala I., Evageliou V. (2022). Physicochemical and Rheological Characteristics of Pectin Extracted from Renewable Orange Peel Employing Conventional and Green Technologies. Food Hydrocoll..

[40] Kute A.B., Mohapatra D., Kotwaliwale N., Giri S.K., Sawant B.P. (2020). Characterization of Pectin Extracted from Orange Peel Powder Using Microwave-Assisted and Acid Extraction Methods. Agric. Res..

[41] Bosch R., Malgas S. (2023). Ultrasound-assisted Enzymatic Extraction of Orange Peel Pectin and Its Characterisation. Int. J. Food Sci. Technol..

[42] Zhou L., Luo J., Xie Q., Huang L., Shen D., Li G. (2023). Dietary Fiber from Navel Orange Peel Prepared by Enzymatic and Ultrasound-Assisted Deep Eutectic Solvents: Physicochemical and Prebiotic Properties. Foods.

[43] Figueira O., Pereira V., Castilho P.C. (2023). A Two-Step Approach to Orange Peel Waste Valorization: Consecutive Extraction of Pectin and Hesperidin. Foods.

[44] Ayala J.R., Montero G., Coronado M.A., García C., Curiel-Alvarez M.A., León J.A., Sagaste C.A., Montes D.G. (2021). Characterization of Orange Peel Waste and Valorization to Obtain Reducing Sugars. Molecules.

[45] Gervasi T., Mandalari G. (2024). Valorization of Agro-Industrial Orange Peel by-Products through Fermentation Strategies. Fermentation.

[46] Fernandes F.A., Heleno S.A., Pinela J., Carocho M., Prieto M.A., Ferreira I.C.F.R., Barros L. (2022). Recovery of Citric Acid from Citrus Peels: Ultrasound-Assisted Extraction Optimized by Response Surface Methodology. Chemosensors.

[47] M’hiri N., Ioannou I., Ghoul M., Boudhrioua N.M., Ayadi S., Chamekh Z., Karmous C., Jallouli S., Ahmed N., Hammami Z. (2015). Proximate Chemical Composition of Orange Peel and Variation of Phenols and Antioxidant Activity during Convective Air Drying. J. New Sci..

[48] Gaind S. (2017). Exploitation of Orange Peel for Fungal Solubilization of Rock Phosphate by Solid State Fermentation. Waste Biomass Valorization.

[49] Mhgub I.M., Hefnawy H.T., Gomaa A.M., Badr H.A. (2018). Chemical Composition, Antioxidant Activity and Structure of Pectin and Extracts from Lemon and Orange Peels. Zagazig J. Agric. Res..

[50] Naqvi S.A.Z., Irfan A., Zaheer S., Sultan A., Shajahan S., Rubab S.L., Ain Q., Acevedo R. (2021). Proximate Composition of Orange Peel, Pea Peel and Rice Husk Wastes and Their Potential Use as Antimicrobial Agents and Antioxidants. Vegetos.

[51] El-Beltagi H.S., Eshak N.S., Mohamed H.I., Bendary E.S.A., Danial A.W. (2022). Physical Characteristics, Mineral Content, and Antioxidant and Antibacterial Activities of Punica Granatum or Citrus Sinensis Peel Extracts and Their Applications to Improve Cake Quality. Plants.

[52] Senit J.J., Velasco D., Gomez Manrique A., Sanchez-Barba M., Toledo J.M., Santos V.E., Garcia-Ochoa F., Yustos P., Ladero M. (2019). Orange Peel Waste Upstream Integrated Processing to Terpenes, Phenolics, Pectin and Monosaccharides: Optimization Approaches. Ind. Crops. Prod..

[53] Iñiguez-Moreno M., Pizaña-Aranda J.J.P., Ramírez-Gamboa D., Ramírez-Herrera C.A., Araújo R.G., Flores-Contreras E.A., Iqbal H.M.N., Parra-Saldívar R., Melchor-Martínez E.M. (2024). Enhancing Pectin Extraction from Orange Peel through Citric Acid-Assisted Optimization Based on a Dual Response. Int. J. Biol. Macromol..

[54] Ferrer V., Paymal N., Quinton C., Tomi F., Luro F. (2022). Investigations of the Chemical Composition and Aromatic Properties of Peel Essential Oils throughout the Complete Phase of Fruit Development for Two Cultivars of Sweet Orange (*Citrus sinensis* (L.) Osb.). Plants.

[55] Farahmandfar R., Tirgarian B., Dehghan B., Nemati A. (2020). Changes in Chemical Composition and Biological Activity of Essential Oil from Thomson Navel Orange (*Citrus sinensis* L. Osbeck) Peel under Freezing, Convective, Vacuum, and Microwave Drying Methods. Food Sci. Nutr..

[56] Singh B., Singh J.P., Kaur A., Yadav M.P. (2021). Insights into the Chemical Composition and Bioactivities of Citrus Peel Essential Oils. Food Res. Int..

[57] Mahato N., Sharma K., Koteswararao R., Sinha M., Baral E., Cho M.H. (2019). Citrus Essential Oils: Extraction, Authentication and Application in Food Preservation. Crit. Rev. Food Sci. Nutr..

[58] Taktak O., Ben Youssef S., Abert Vian M., Chemat F., Allouche N. (2021). Physical and Chemical Influences of Different Extraction Techniques for Essential Oil Recovery from Citrus Sinensis Peels. J. Essent. Oil Bear. Plants.

[59] Bustamante J., van Stempvoort S., García-Gallarreta M., Houghton J.A., Briers H.K., Budarin V.L., Matharu A.S., Clark J.H. (2016). Microwave Assisted Hydro-Distillation of Essential Oils from Wet Citrus Peel Waste. J. Clean. Prod..

[60] Hilali S., Fabiano-Tixier A.-S., Ruiz K., Hejjaj A., Ait Nouh F., Idlimam A., Bily A., Mandi L., Chemat F. (2019). Green Extraction of Essential Oils, Polyphenols, and Pectins from Orange Peel Employing Solar Energy: Toward a Zero-Waste Biorefinery. ACS Sustain. Chem. Eng..

[61] Youcef-Ettoumi K., Zouambia Y., Moulai-Mostefa N. (2021). Chemical Composition, Antimicrobial and Antioxidant Activities of Algerian Citrus Sinensis Essential Oil Extracted by Hydrodistillation Assisted by Electromagnetic Induction Heating. J. Food Sci. Technol..

[62] Golmohammadi M., Borghei A., Zenouzi A., Ashrafi N., Taherzadeh M.J. (2018). Optimization of Essential Oil Extraction from Orange Peels Using Steam Explosion. Heliyon.

[63] Shehata M.G., Awad T.S., Asker D., El Sohaimy S.A., Abd El- Aziz N.M., Youssef M.M. (2021). Antioxidant and Antimicrobial Activities and UPLC-ESI-MS/MS Polyphenolic Profile of Sweet Orange Peel Extracts. Curr. Res. Food Sci..

[64] Lux P.E., Carle R., Zacarías L., Rodrigo M.-J., Schweiggert R.M., Steingass C.B. (2019). Genuine Carotenoid Profiles in Sweet Orange [*Citrus sinensis* (L.) Osbeck Cv. Navel] Peel and Pulp at Different Maturity Stages. J. Agric. Food Chem..

[65] Magalhães D., Gonçalves R., Rodrigues C.V., Rocha H.R., Pintado M., Coelho M.C. (2024). Natural Pigments Recovery from Food By-Products: Health Benefits towards the Food Industry. Foods.

[66] Singh B., Singh J.P., Kaur A., Singh N. (2020). Phenolic Composition, Antioxidant Potential and Health Benefits of Citrus Peel. Food Res. Int..

[67] Anastas P.T., Zimmerman J.B. (2003). Peer Reviewed: Design through the 12 Principles of Green Engineering 2003. Environ. Sci. Technol..

[68] Liu N., Li X., Zhao P., Zhang X., Qiao O., Huang L., Guo L., Gao W. (2021). A Review of Chemical Constituents and Health-Promoting Effects of Citrus Peels. Food Chem..

[69] Teigiserova D.A., Tiruta-Barna L., Ahmadi A., Hamelin L., Thomsen M. (2021). A Step Closer to Circular Bioeconomy for Citrus Peel Waste: A Review of Yields and Technologies for Sustainable Management of Essential Oils. J. Environ. Manag..

[70] Khandare R.D., Tomke P.D., Rathod V.K. (2021). Kinetic Modeling and Process Intensification of Ultrasound-Assisted Extraction of d-Limonene Using Citrus Industry Waste. Chem. Eng. Process.-Process Intensif..

[71] Karanicola P., Patsalou M., Stergiou P.Y., Kavallieratou A., Evripidou N., Christou P., Panagiotou G., Damianou C., Papamichael E.M., Koutinas M. (2021). Ultrasound-Assisted Dilute Acid Hydrolysis for Production of Essential Oils, Pectin and Bacterial Cellulose via a Citrus Processing Waste Biorefinery. Bioresour. Technol..

[72] Ciriminna R., Fidalgo A., Delisi R., Carnaroglio D., Grillo G., Cravotto G., Tamburino A., Ilharco L.M., Pagliaro M. (2017). High-Quality Essential Oils Extracted by an Eco-Friendly Process from Different Citrus Fruits and Fruit Regions. ACS Sustain. Chem. Eng..

[73] Aboudaou M., Ferhat M.A., Hazzit M., Ariño A., Djenane D. (2019). Solvent Free-Microwave Green Extraction of Essential Oil from Orange Peel (*Citrus sinensis* L.): Effects on Shelf Life of Flavored Liquid Whole Eggs during Storage under Commercial Retail Conditions. J. Food Meas. Charact..

[74] de Miera B.S., Cañadas R., González-Miquel M., González E.J. (2023). Recovery of Phenolic Compounds from Orange Peel Waste by Conventional and Assisted Extraction Techniques Using Sustainable Solvents. Front. Biosci. Elite.

[75] Mussatto S.I., Ballesteros L.F., Martins S., Teixeira J.A. (2011). Extraction of Antioxidant Phenolic Compounds from Spent Coffee Grounds. Sep. Purif. Technol..

[76] Boeing J.S., Barizão É.O., e Silva B.C., Montanher P.F., de Cinque Almeida V., Visentainer J.V. (2014). Evaluation of Solvent Effect on the Extraction of Phenolic Compounds and Antioxidant Capacities from the Berries: Application of Principal Component Analysis. Chem. Cent. J..

[77] Saini A., Panesar P.S., Bera M.B. (2019). Comparative Study on the Extraction and Quantification of Polyphenols from Citrus Peels Using Maceration and Ultrasonic Technique. Curr. Res. Nutr. Food Sci..

[78] Boukroufa M., Boutekedjiret C., Petigny L., Rakotomanomana N., Chemat F. (2015). Bio-Refinery of Orange Peels Waste: A New Concept Based on Integrated Green and Solvent Free Extraction Processes Using Ultrasound and Microwave Techniques to Obtain Essential Oil, Polyphenols and Pectin. Ultrason. Sonochem..

[79] Leo C.H., Foo S.Y., Tan J.C.W., Tan U.-X., Chua C.K., Ong E.S. (2022). Green Extraction of Orange Peel Waste Reduces TNFα-Induced Vascular Inflammation and Endothelial Dysfunction. Antioxidants.

[80] Barrales F.M., Silveira P., de Paula Menezes Barbosa P., Ruviaro A.R., Paulino B.N., Pastore G.M., Macedo G.A., Martinez J. (2018). Recovery of Phenolic Compounds from Citrus By-Products Using Pressurized Liquids—An Application to Orange Peel. Food Bioprod. Process..

[81] Gómez-Urios C., Viñas-Ospino A., Puchades-Colera P., López-Malo D., Frígola A., Esteve M.J., Blesa J. (2022). Sustainable Development and Storage Stability of Orange By-Products Extract Using Natural Deep Eutectic Solvents. Foods.

[82] Wang Z., Mei X., Chen X., Rao S., Ju T., Li J., Yang Z. (2023). Extraction and Recovery of Bioactive Soluble Phenolic Compounds from Brocade Orange (*Citrus sinensis*) Peels: Effect of Different Extraction Methods Thereon. LWT.

[83] Meneguzzo F., Brunetti C., Fidalgo A., Ciriminna R., Delisi R., Albanese L., Zabini F., Gori A., dos Santos Nascimento L.B., De Carlo A. (2019). Real-Scale Integral Valorization of Waste Orange Peel via Hydrodynamic Cavitation. Processes.

[84] Shahram H., Dinani S.T., Amouheydari M. (2019). Effects of Pectinase Concentration, Ultrasonic Time, and PH of an Ultrasonic-Assisted Enzymatic Process on Extraction of Phenolic Compounds from Orange Processing Waste. J. Food Meas. Charact..

[85] Savic Gajic I.M., Savic I.M., Gajic D.G., Dosic A. (2021). Ultrasound-Assisted Extraction of Carotenoids from Orange Peel Using Olive Oil and Its Encapsulation in ca-Alginate Beads. Biomolecules.

[86] Montero-Calderon A., Cortes C., Zulueta A., Frigola A., Esteve M.J. (2019). Green Solvents and Ultrasound-Assisted Extraction of Bioactive Orange (*Citrus sinensis*) Peel Compounds. Sci. Rep..

[87] Toprakçı İ., Kurtulbaş E., Pekel A.G., Şahin S. (2021). Application of D--optimal Design for Automatic Solvent Extraction of Carotenoid from Orange Peel. J. Food Process. Preserv..

[88] Terlidis K., Athanasiadis V., Chatzimitakos T., Bozinou E., Lalas S.I. (2023). Carotenoids Extraction from Orange Peels Using a Thymol-Based Hydrophobic Eutectic Solvent. AppliedChem.

[89] Jiao Y., Li D., Chang Y., Xiao Y. (2018). Effect of Freeze-thaw Pretreatment on Extraction Yield and Antioxidant Bioactivity of Corn Carotenoids (Lutein and Zeaxanthin). J. Food Qual..

[90] Ignaczak A., Salamon A., Kowalska J., Marzec A., Kowalska H. (2023). Influence of Pre-Treatment and Drying Methods on the Quality of Dried Carrot Properties as Snacks. Molecules.

[91] Boukroufa M., Boutekedjiret C., Chemat F. (2017). Development of a Green Procedure of Citrus Fruits Waste Processing to Recover Carotenoids. Resour. Effic. Technol..

[92] Suthar P., Kaushal M., Vaidya D., Thakur M., Chauhan P., Angmo D., Kashyap S., Negi N. (2023). Deep Eutectic Solvents (DES): An Update on the Applications in Food Sectors. J. Agric. Food Res..

[93] Riyamol, Gada Chengaiyan J., Rana S.S., Ahmad F., Haque S., Capanoglu E. (2023). Recent Advances in the Extraction of Pectin from Various Sources and Industrial Applications. ACS Omega.

[94] Naqash F., Masoodi F.A., Rather S.A., Wani S.M., Gani A. (2017). Emerging Concepts in the Nutraceutical and Functional Properties of Pectin—A Review. Carbohydr. Polym..

[95] Saberian H., Hamidi-Esfahani Z., Ahmadi Gavlighi H., Banakar A., Barzegar M. (2018). The Potential of Ohmic Heating for Pectin Extraction from Orange Waste. J. Food Process. Preserv..

[96] Yang N., Jin Y., Tian Y., Jin Z., Xu X. (2016). An Experimental System for Extraction of Pectin from Orange Peel Waste Based on the O-Core Transformer Structure. Biosyst. Eng..

[97] Waheed A., Akram S., Ashraf R., Mushtaq M., Adnan A. (2020). Kinetic Model and Optimization for Enzyme-assisted Hydrodistillation of D-limonene-rich Essential Oil from Orange Peel. Flavour Fragr. J..

[98] Liu K., Deng W., Hu W., Cao S., Zhong B., Chun J. (2019). Extraction of ‘Gannanzao’ Orange Peel Essential Oil by Response Surface Methodology and Its Effect on Cancer Cell Proliferation and Migration. Molecules.

[99] Argun M.E., Argun M.Ş., Arslan F.N., Nas B., Ates H., Tongur S., Cakmakcı O. (2022). Recovery of Valuable Compounds from Orange Processing Wastes Using Supercritical Carbon Dioxide Extraction. J. Clean. Prod..

[100] Lachos-Perez D., Baseggio A.M., Mayanga-Torres P.C., Junior M.R.M., Rostagno M.A., Martínez J., Forster-Carneiro T. (2018). Subcritical Water Extraction of Flavanones from Defatted Orange Peel. J. Supercrit. Fluids.

[101] Murador D.C., Mesquita L.M.D.S., Neves B.V., Braga A.R.C., Martins P.L.G., Zepka L.Q., De Rosso V. (2021). V Bioaccessibility and Cellular Uptake by Caco-2 Cells of Carotenoids and Chlorophylls from Orange Peels: A Comparison between Conventional and Ionic Liquid Mediated Extractions. Food Chem..

[102] Murador D.C., Salafia F., Zoccali M., Martins P.L.G., Ferreira A.G., Dugo P., Mondello L., de Rosso V.V., Giuffrida D. (2019). Green Extraction Approaches for Carotenoids and Esters: Characterization of Native Composition from Orange Peel. Antioxidants.

[103] Murador D.C., Braga A.R.C., Martins P.L.G., Mercadante A.Z., de Rosso V.V. (2019). Ionic Liquid Associated with Ultrasonic-Assisted Extraction: A New Approach to Obtain Carotenoids from Orange Peel. Food Res. Int..

[104] Viñas-Ospino A., Panić M., Radojčić- Redovniković I., Blesa J., Esteve M.J. (2023). Using Novel Hydrophobic Deep Eutectic Solvents to Improve a Sustainable Carotenoid Extraction from Orange Peels. Food Biosci..

[105] Kamal M.M., Kumar J., Mamun M.A.H., Ahmed M.N.U., Shishir M.R.I., Mondal S.C. (2021). Extraction and Characterization of Pectin from Citrus Sinensis Peel. J. Biosyst. Eng..

[106] Jokić S., Molnar M., Cikoš A.-M., Jakovljević M., Šafranko S., Jerković I. (2020). Separation of Selected Bioactive Compounds from Orange Peel Using the Sequence of Supercritical CO2 Extraction and Ultrasound Solvent Extraction: Optimization of Limonene and Hesperidin Content. Sep. Sci. Technol..

[107] Angoy A., Ginies C., Goupy P., Bornard I., Ginisty P., Sommier A., Valat M., Chemat F. (2020). Development of a Green Innovative Semi-Industrial Scale Pilot Combined Microwave Heating and Centrifugal Force to Extract Essential Oils and Phenolic Compounds from Orange Peels. Innov. Food Sci. Emerg. Technol..

[108] Tsouko E., Maina S., Ladakis D., Kookos I.K., Koutinas A. (2020). Integrated Biorefinery Development for the Extraction of Value-Added Components and Bacterial Cellulose Production from Orange Peel Waste Streams. Renew Energy.

[109] Tovar A.K., Godínez L.A., Espejel F., Ramírez-Zamora R.-M., Robles I. (2019). Optimization of the Integral Valorization Process for Orange Peel Waste Using a Design of Experiments Approach: Production of High-Quality Pectin and Activated Carbon. Waste Manag..

[110] Vaez S., Karimi K., Mirmohamadsadeghi S., Jeihanipour A. (2021). An Optimal Biorefinery Development for Pectin and Biofuels Production from Orange Wastes without Enzyme Consumption. Process Saf. Environ. Prot..

[111] Martins M., Goldbeck R. (2023). Integrated Biorefinery for Xylooligosaccharides, Pectin, and Bioenergy Production from Orange Waste. Biofuels Bioprod. Biorefining.

